# Associations between Dietary Patterns and Cardiometabolic Risks in Japan: A Cross-Sectional Study from the Fukushima Health Management Survey, 2011–2015

**DOI:** 10.3390/nu12010129

**Published:** 2020-01-02

**Authors:** Enbo Ma, Tetsuya Ohira, Akira Sakai, Seiji Yasumura, Atsushi Takahashi, Junichiro Kazama, Michio Shimabukuro, Hironori Nakano, Kanako Okazaki, Masaharu Maeda, Hirooki Yabe, Yuriko Suzuki, Kenji Kamiya

**Affiliations:** 1 Health Promotion Center, Fukushima Medical University, 1 Hikariga-oka, Fukushima 960-1295, Japan; teoohira@fmu.ac.jp; 2 Department of Epidemiology, Fukushima Medical University School of Medicine, 1 Hikariga-oka, Fukushima 960-1295, Japan; h-nakano@fmu.ac.jp (H.N.); kana-oka@fmu.ac.jp (K.O.); 3 Radiation Medical Science Center for Fukushima Health Management Survey, Fukushima Medical University, 1 Hikariga-oka, Fukushima 960-1295, Japan; sakira@fmu.ac.jp (A.S.); yasumura@fmu.ac.jp (S.Y.); junior@fmu.ac.jp (A.T.); jjkaz@fmu.ac.jp (J.K.); shima01@fmu.ac.jp (M.S.); masagen@fmu.ac.jp (M.M.); kkamiya@fmu.ac.jp (K.K.); 4 Department of Radiation Life Sciences, Fukushima Medical University School of Medicine, 1 Hikariga-oka, Fukushima 960-1295, Japan; 5 Department of Public Health, Fukushima Medical University School of Medicine, 1 Hikariga-oka, Fukushima 960-1295, Japan; 6 Department of Gastroenterology, Fukushima Medical University School of Medicine, 1 Hikariga-oka, Fukushima 960-1295, Japan; 7 Department of Nephrology and Hypertension, Fukushima Medical University School of Medicine, 1 Hikariga-oka, Fukushima 960-1295, Japan; 8 Department of Diabetes, Endocrinology and Metabolism School of Medicine, Fukushima Medical University, 1 Hikariga-oka, Fukushima 960-1295, Japan; 9 Department of Disaster Psychiatry, Fukushima Medical University School of Medicine, 1 Hikariga-oka, Fukushima 960-1295, Japan; 10 Department of Neuropsychiatry, Fukushima Medical University School of Medicine, 1 Hikariga-oka, Fukushima 960-1295, Japan; hyabe@fmu.ac.jp; 11 Department of Adult Mental Health, National Institute of Mental Health, National Center of Neurology and Psychiatry, Tokyo 187-8553, Japan; yrsuzuki@gmail.com

**Keywords:** dietary pattern, food frequency questionnaire, cardiometabolic risk, Fukushima Health Management Survey

## Abstract

Cardiometabolic risks were increasing in Fukushima residents after the Great East Japan Earthquake. We examined the association between dietary patterns and cardiometabolic risks in those aged ≥16 years. Dietary patterns were derived by principal component analysis for participants who underwent at least one diet assessment using a short-form food frequency questionnaire during 2011–2013 and a health checkup in 2014 and 2015 (*n* = 15,409 and 14,999, respectively). In 2014, the adjusted prevalence ratio (PR) and 95% confidence interval (CI) in the highest versus lowest quartile of accumulative mean scores were 0.97 (0.96–0.99) for overweight/obesity, 0.96 (0.95–0.97) for total cholesterol (TC) ≥ 220 mg/dL, 0.96 (0.95–0.98) for low-density lipoprotein cholesterol (LDL-C) ≥ 140 mg/dL, and 0.97 (0.96–0.99) for triglycerides ≥ 150 mg/dL for a vegetable diet and 1.03 (1.01–1.04) for TC ≥ 220 mg/dL and 1.02 (1.01–1.04) for LDL-C ≥ 140 mg/dL for a juice/milk diet. In 2015, we found consistently significant associations for the vegetable and juice/milk diets, and the PR and 95% CI were 0.99 (0.98–1.00) for HDL-C < 40 mg/dL for a meat diet. The continuous promotion of the vegetable pattern diet is necessary to reduce cardiometabolic risks, particularly dyslipidemia, in Japan.

## 1. Introduction

With a worldwide shift in dietary patterns, the current health status of the population has also shifted to a “pandemic” of obesity and increased cardiometabolic risks [1]. The Great East Japan Earthquake in March 2011 affected the health status of the residents in the disaster areas, with increased cardiometabolic risks, such as overweight/obesity, hypertension, diabetes mellitus, and dyslipidemia, being reported [2,3,4]. The prevalence of hypertension peaked one year after the disaster and showed a decline tendency [4]; the body weight/waist circumference was increasing with a deterioration of the high-density lipoprotein cholesterol level among relocated survivors even more than one year post-disaster [3]. Currently, ischemic heart disease in Fukushima has the highest prevalence in Japan, being twice that of the national average. However, the association between diet, the most important, modifiable risk factor, and increased cardiometabolic risks after the disaster has not been fully investigated yet.

Changes in nutrient intake might be difficult to evaluate and reflect over time. Studies on dietary patterns more resembling actual eating behaviors and focusing on multiple food groups have methodological advantages compared to studies based on a single dietary product or nutrient [5,6,7]. One reason is that some highly correlative or interactive nutrients might have strong enough effects to be detected [5,6,7]. Therefore, foods eaten in combination are more suitable for learning about people’s dietary behaviors [5]. 

Controlling and modifying lifestyle risk factors is effective in maintaining good health. A high intake of fruits and vegetables, salads, rice, chicken, fish, cereals, and low-fat dairy products appears to be effective in lowering blood pressure (BP) [8,9] and glycated hemoglobin levels [10]. Snacks increase the risk of hypercholesterolemia [11], while a Mediterranean diet [12,13] and Dietary Approaches to Stop Hypertension (DASH) [14] are inversely associated with metabolic syndrome (MetS). However, few studies have reported on a relationship between dietary patterns and hypertension, glucose intolerance, blood lipid profiles, and MetS in Japanese populations, which are mainly found in middle-aged and older people [10,15,16,17]. In this study, we hypothesized that stable dietary patterns might affect late health conditions in adults. We reported dietary patterns identified using the Fukushima Health Management Survey (FHMS) between 2011 and 2013 and the associations between dietary patterns and cardiometabolic risks in residents of Fukushima, Japan aged more than 16 years old in 2014 and 2015. This would be the first comprehensive study to assess the dietary pattern and to investigate the associations between adhering to dietary status and broad cardiometabolic risks in a population from the disaster area since 11 March 2011.

## 2. Materials and Methods

### 2.1. Study Participants

The FHMS was initiated in 2011 after the Great East Japan Earthquake. The target population comprised 210,189 residents living in the evacuation zones along the radiation disclosure areas. Overall, 88,613 participants (42.2%) responded to the survey questionnaires in the 2011 fiscal year. The details of the study protocol and baseline profiles were described earlier [18]. We used data of individuals aged ≥16 years from the Mental Health and Lifestyle Survey 2011, 2012, and 2013, conducted as part of the FHMS and including a self-administrated questionnaire on social and demographics, medical history, and lifestyle and a food frequency questionnaire (FFQ) (*n* = 172,664). This study was approved by the Committee for Ethics at Fukushima Medical University, Japan (nos. 1316, 1319, and 29064).

### 2.2. Dietary Intake Assessment

We used a short-form FFQ with 19 food items to determine the food intake during the 6 months preceding the survey date. The FFQ was a valid and modified version of the Hiroshima and Nagasaki Life Span Study [19]. We divided the 19 food items into 8 food groups: non-juice fruit/vegetable (fruits, green vegetables, red and orange vegetables, and light-colored vegetables), fruit/vegetable juice, meat subgroup (chicken, beef/pork, and ham/sausages), soybean product (fermented soybean, soy milk, miso soup, tofu, and boiled beans), fish (raw and cooked), and dairy (milk, yogurt, and lactobacillus drinks). We asked the participants how often they consumed individual food items, with six response choices for frequency: none, <1 time/week, 1–2 times/week, 3–4 times/week, 5–6 times/week, or every day.

### 2.3. End-Point Determination

We retrieved data from the comprehensive health checkups conducted in 2014 and 2015 as part of the FHMS and including cardiometabolic factors: overweight/obesity, hypertension, fasting blood glucose, hemoglobin A1c (HbA1c1), triglycerides, low-density lipoprotein cholesterol (LDL-C), high-density lipoprotein cholesterol (HDL-C), and MetS.

We defined the cardiometabolic factors as follows: overweight as body mass index (BMI) ≥ 25 kg/m^2^; hypertension as systolic blood pressure (SBP) ≥ 140 mmHg, diastolic blood pressure (DBP) ≥ 90 mmHg, or the use of antihypertensive medication; high fasting blood glucose as fasting plasma glucose ≥ 126 mg/dL; and glucose intolerance as HbA1c1 ≥ 6.5%. Participants who met the following criteria were diagnosed with dyslipidemia: HDL-C < 40 mg/dL, LDL-C ≥ 140 mg/dL, total cholesterol [TC] ≥ 220 mg/dL, or high triglycerides ≥ 150 mg/dL, while MetS was defined according to the Japanese Diabetes Association guidelines of 2005: waist circumstance ≥ 85 cm in males and ≥90 cm in females, plus two or more of triglycerides ≥ 150 mg/dL, HDL-C < 40 mg/dL, SBP ≥ 130 mmHg or DBP ≥ 85 mmHg, and raised fasting blood glucose ≥ 110 mg/dL. 

### 2.4. Statistical Analysis

In the Mental Health Surveys, FFQs were available only in 2011, 2012, and 2013. We excluded pregnant women (*n* = 1793) and those with cerebrovascular and cardiovascular disease or cancer, currently or historically (*n* = 33,269). In all, 18,173 and 17,973 individuals underwent health checkups in 2014 and 2015, respectively. We analyzed those aged ≥16 years who underwent at least one wave of FFQ diet assessment in 2011, 2012, or 2013 and underwent a health checkup in 2014 or 2015. For those who did not answer some dietary questions (13.0% missed one and 4.4% missed two questions in the 2014 dataset, while 13.2% missed one and 4.4% missed two questions in the 2015 dataset), we replaced the missing values by using the median value of frequency for that food item by survey year and sex [20]. Finally, we enrolled 15,409 participants in 2014 and 14,999 participants in 2015 with dietary and health checkup data into this study (Figure S1 in Supplementary Materials). For the frequency of dietary intake of each food group, we used the daily midpoint for the frequency category, for example, we assessed ‘3–4 times/week’ as 0.5 times/day [20].

We derived dietary patterns and considered the primary independent variables across the three waves (2011–2013) from 19 food items without alcohol consumption using principal component analysis (PCA). We used Varimax rotation with the identified dietary patterns to improve their interpretability. We selected factor numbers mainly according to eigenvalues > 1.5, scree plots, and factor interpretability and considered food items with absolute factor loadings ≥0.3 to account for each component [21]. We labeled the derived dietary patterns as vegetable, juice/milk, and meat on the basis of food items with high factor loadings on each dietary pattern. The eigenvalues of the vegetable, juice/milk, and meat dietary patterns were 4.191, 1.751, and 1.571, respectively, in the 2014 dataset and 4.159, 1.782, and 1.559, respectively, in the 2015 dataset. The cumulative variance explained was 39.54% in the 2014 dataset and 39.47% in the 2015 dataset. Cronbach’s alpha coefficient for each dietary pattern indicated a higher internal reliability of these measures: 0.798 for vegetable, 0.810 for juice/milk, and 0.815 for meat in the 2014 dataset and 0.796 for vegetable, 0.809 for juice/milk, and 0.813 for meat in the 2015 dataset. We derived almost the same factors from PCA by sex; therefore, we only reported dietary patterns for the total participants.

At each wave, we assigned the participants pattern-specific dietary pattern scores, which we calculated as the sum of the products of the factor loading coefficients and standardized intake of food items. Higher scores represent a closer resemblance of a participant’s diet to the identified pattern [22]. We also calculated the mean of the cumulative dietary pattern scores of each participant for each dietary pattern in order to better reflect the long-term diet and reduce the dietary measurement error [23]. Finally, we categorized the accumulative means of dietary pattern scores in 2014 and 2015 into quartiles.

With regard to the covariates at the baseline for multivariable adjustment, we selected the first available value of a variable if an individual had participated in more than two diet surveys between 2011 and 2013. We considered the potential confounding factors mainly based on the previous publications of FHMS [4,20]. We classified the education status into elementary, junior high, high school, vocational university, and university and above; smoking history into never, former, and current; and alcohol consumption into never, occasional, and regular. As there were no questions on smoking history and alcohol consumption for participants aged between 16 and 19 years, we assigned a unique category for these two variables in order to include the participants in the multivariate analysis. We also grouped physical activity into none, 1 time/week, 2–4 times/week, and every day and resident places post-earthquake into living in a shelter or temporary house, an apartment or rental house, and at relatives’ or in one’s own house. We measured depression using the Japanese version of the Kessler Psychological Distress Scale (K6), with higher scores signifying a worse mental health status (range: 0–24), grouped as low (K6 < 13) or high (K6 ≥ 13) [20].

We tested the differences between the social, demographic and cardiometabolic risk proportions using the chi-square test and the differences in health checkup values using nonparametric analysis of variance (ANOVA) across quartile categories. We used Poisson regression with robust error variance to derive prevalence ratios (PRs) and 95% confidence intervals (Cis) to measure the associations between the means of the dietary pattern scores (with the first quartile as the reference) and each cardiometabolic risk, adjusted for age (continuous), sex, education, smoking history, alcohol consumption, physical activity, change of residence, and depression status. We assess the linear trend across quartiles by assigning the median value of dietary pattern score as a continuous variable. We analyzed all data using SAS statistical software ver. 9.4 for Windows (SAS Institute, Cary, NC, USA). All *p*-values reported were two-sided, and *p* < 0.05 was considered statistically significant.

### 2.5. Sensitivity Analysis

We conducted the first sensitivity analysis for 9674 participants in 2014 and 9241 participants in 2015 to derive dietary patterns and test associations; these individuals had undergone all three waves of diet assessment. We conducted the second sensitivity analysis after removing the individuals hospitalized or taking medicine for hypertension, diabetes, or hyperlipidemia, to test the associations for hypertension (*n* = 9692 in 2014; *n* = 9336 in 2015), high glucose and HbA1c (*n* = 13,990 in 2014; *n* = 13,557 in 2015), and dyslipidemia (*n* = 11,555 in 2014; *n* = 11,007 in 2015). Excluding the individuals with all three conditions, we had 6350 subjects in 2014 and 6002 subjects in 2015 for testing associations for the BMI and MetS.

## 3. Results

**Figure 1:**
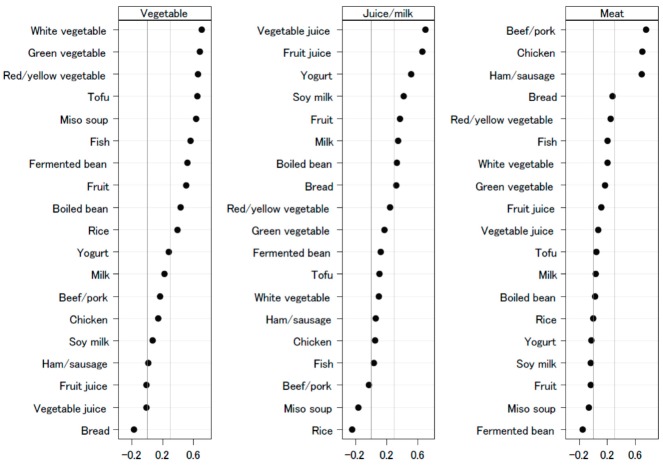
Dietary patterns loadings by food groups, Fukushima Health Management Survey, 2011–2013.

Figure 1 shows the dietary patterns with food group loadings graphically. The first dietary pattern, named ‘vegetable’, was positively loaded for food groups of vegetables, fish, fruits, bean products (tofu, fermented beans, boiled beans, and miso soup), and rice and negatively loaded with fruit juice, vegetable juice, and bread. The second dietary pattern, named ‘juice/milk’, was positively loaded for groups of vegetable juice, fruit juice, yogurt, soymilk, fruits, milk, boiled beans, and bread and negatively loaded for beef/pork, miso soup, and rice. Meanwhile, the third dietary pattern, named ‘meat’, was positively loaded for chicken, beef/pork, and ham/sausage and negatively loaded for yogurt, soymilk, fruit, miso soup, and fermented bean.

The characteristics of the participants in the 2014 and 2015 datasets were very similar. Table 1, Table 2 and Table 3 show the social and demographic characteristics of participants at the baseline for 2011, 2012, and 2013 and their health checkup details of 2014 and 2015 according to the quartiles of each dietary pattern score. The median age of all participants was 62 years. Women were the domain participants. Participants with a higher education level were less likely to consume the vegetable pattern diet but more likely to consume the juice/milk and meat pattern diets (*p*-values < 0.001). Participants with frequent physical practices were more likely to consume the vegetable and juice/milk pattern diets but were less likely to consume the meat pattern diet (*p*-values < 0.001). Participants were less likely to consume the vegetable pattern diet if they were living in shelters/temporary houses/rental houses (*p* < 0.001).

The SBP level, hypertension proportion, and fasting blood glucose level were higher in participants with higher vegetable dietary pattern scores but were lower in participants with higher meat dietary pattern scores. The proportion of LDL-C ≥ 140 mg/dL and triglycerides ≥ 150 mg/dL declined along ascendant quartiles of the vegetable and meat dietary pattern scores. TC and LDL-C levels showed similar decreased trends in the vegetable dietary pattern and increased trends in the juice/milk dietary pattern. The proportion of hypertriglyceridemia decreased with an increase in vegetable and juice/milk dietary pattern scores. The HDL-C level was positively correlated with juice/milk dietary pattern scores, while the MetS proportion was negatively correlated with the meat dietary pattern scores.

The cumulative mean score of three dietary patterns during 2011–2013 was compared by cardiometabolic risks in 2014 (Table S1). In this univariate analysis, the vegetable pattern score was significantly lower in those with overweight, TC ≥ 220 mg/dL, LDL-C ≥ 140 mg/dL, and triglycerides ≥ 150 mg/dL, but was higher in those with hypertension, fasting blood glucose ≥ 126 mg/dL, and HbA1c1 ≥ 6.5% compared with the corresponding counterpart (*p*-values < 0.05). The juice/milk pattern score was significantly lower in those with overweight, HDL-C < 40 mg/dL, triglycerides ≥ 150 mg/dL, and MetS, but higher in those with TC ≥ 220 mg/dL and LDL-C ≥ 140 mg/dL (*p*-values < 0.05). Except for LDL-C ≥ 140 mg/dL, the meat pattern score was significantly higher in those with cardiometabolic risk (*p*-values < 0.05). Similar results were observed in 2015 (Table S2).

Table 4 and Table 5 show the associations between accumulative dietary pattern mean scores and metabolic risk with multivariable adjustment. In 2014 (Table 4), the vegetable dietary pattern (Model 2) was inversely associated with overweight, hypertension, TC ≥ 220 mg/dL, LDL-C ≥ 140 mg/dL, triglycerides ≥ 150 mg/dL, and MetS, with significant decreasing trends. Participants in the highest quartile, compared with the lowest quartile, of vegetable pattern scores had a 2–4% reduction of these cardiometabolic risks. The first sensitivity analysis showed significantly inverse associations in participants with all three waves of available FFQs. The second sensitivity analysis showed significant but not persistent associations of vegetable dietary pattern scores with overweight, hypertension, and MetS.

In addition, we observed a significantly positive association of TC ≥ 220 mg/dL and LDL-C ≥ 140 mg/dL with the juice/milk dietary pattern. Those in the highest quartile compared with those in the lowest quartile of juice/milk patterns scores had a 2–3% increased risk. The first sensitivity analysis showed a significantly positive association of TC ≥ 220 mg/dL, while the second sensitivity analysis showed a significantly positive association of impaired blood glucose control, TC ≥ 220 mg/dL, and LDL-C ≥ 140 mg/dL. 

In 2015 (Table 5), the vegetable dietary pattern was inversely associated with overweight, hypertension, dyslipidemia, and MetS. Those in the highest quartile compared with those in the lowest quartile of vegetable pattern scores had a 1–4% reduction of these cardiometabolic risks (Model 2). The second sensitivity analysis showed no significance of overweight, hypertension and MetS and a significant inverse association of the vegetable dietary pattern with high blood glucose and impaired blood glucose control. In 2015, sensitivity analysis showed that the juice/milk dietary pattern scores were positively associated with TC ≥ 220 mg/dL and LDL-C ≥ 140 mg/dL. We also observed an inverse association of the meat dietary pattern with HDL-C < 40 mg/dL and triglycerides ≥ 150 mg/dL; in the second sensitivity analysis, the significance remained for HDL-C < 40 mg/dL.

Participants with a family history of stroke, heart disease, diabetes, and cancer constituted 23.5%, 19.2%, 15.3%, and 32.1%, respectively, of the populace in 2014 and 25.7%, 19.3%, 15.6%, and 32.4%, respectively, of the populace in 2015. We repeated the above multivariate analysis with added dummy variables of the family history of stroke, heart disease, diabetes, and/or cancer for further adjustments; the statistically significant PRs and 95% CIs remained for each cardiometabolic risk factor in 2014 and 2015 (data not shown).

We also conducted stratified analysis by age group (≥45 years), sex, weight, smoking history, and alcohol consumption, but the significant associations between the accumulative dietary pattern mean scores and the cardiometabolic risk factors did not change (data not shown).

## 4. Discussion

In this large population-based prospective study, we observed significantly inverse associations between the vegetable dietary pattern and cardiometabolic risks, including overweight and dyslipidemia, significantly positive associations between the juice/milk dietary pattern and high TC and high LDL-C concentrations, and significantly inverse associations between the meat dietary pattern and low HDL-C concentration. To our knowledge, this is the first study to comprehensively examine the association of cardiometabolic risks with dietary patterns by longitudinal surveys in Japanese populations. Considering the long-term effects of diets on these cardiometabolic factors, the vegetable pattern diets might reduce the risk of dyslipidemia prominently.

The dietary patterns identified in this study were similar to those in other Japanese studies; for example, the National Health and Nutrition Survey in 2012 (vegetable, high-bread and low-rice, and high-meat and low-fish dietary patterns) [10], the Jichi Medical School Cohort Study (vegetable, western, and meat dietary patterns) [15], the Ohsaki Cohort Study (Japanese, high-dairy, and animal food dietary patterns) [24], and the Japan Collaborative Cohort Study (vegetable, dairy products, and animal food dietary patterns) [25].

The vegetable dietary pattern in our study had similar characteristics of high intake in the healthy/prudent dietary patterns that were most reproducible in Japanese [26,27,28] and other ethnicities [29,30,31]. Japanese and Mediterranean diets have similar features of customarily eating seafood, vegetables, and fruits, and instead of nuts that are commonly eaten in Western countries, soybean and soy products are popular among the Japanese [13,32]. A recent systematic review reported that the top three categories of the Japanese diet are soybean/soybean-derived products, seafood, and vegetables, plus rice and miso soup [33], very similar to our study.

For the vegetable dietary pattern, our results were consistent with other studies with regard to decreased risk of obesity [28], low HDL-C concentration [15], and high LDL-C concentration [34]. A Japanese study reported an inverse association of MetS and all its components with the intake of folate, dietary fiber, carotene, iron, vitamin C, and potassium, which are abundant in vegetables and fruits [17]. We obtained similar results regarding the inverse association of MetS and all its components with the risk of hypertension [15,16] and diabetes [35] in the main analysis (Model 2). However, for hypertension, significant associations with vegetable and juice/milk dietary patterns did not remain in the second sensitivity analysis, which might be because 35% of participants with hypertension were in treatment and then on a cautious diet [36]. The prevalence of using antihypertensive drugs in middle-aged populations regardless of the evacuation status were continuously increasing between 2008 and 2014 [4]. In contrast, those who know they had hypertension but were not on medication might relate to an increase in the consumption of vegetables [15]. Hypertension might also be considered as being partially mediated by obesity [17] or sodium [37]; however, we did not observe significant associations in the analysis by adding the BMI for adjustment, and data for sodium or the sodium–potassium ratio were not available. Similar to hypertension, a lack of significant associations in the subgroup analysis for MetS, with a much reduced sample size, needs further investigation.

When excluding participants with diabetes, we observed that the risk of high blood glucose and impaired blood glucose control (2015) was significantly associated with the vegetable dietary pattern. A recent study in a Korean population reported that a diet rich in vegetables, mushrooms, seaweeds, fruits, and soy products and low in fatty fish and high-fat meat might potentially play a protective role in type 2 diabetes development [38]. A Chinese study showed a decrease in the risk of impaired blood glucose control by a similar healthy food pattern [39]. Soybean foods contain folate, which decreases homocysteine and the risk of other coronary heart diseases and type 2 diabetes [40]. The combination of beans and rice has been found to be protective against obesity among Brazilian adults [41]. The Japanese dietary pattern is reportedly associated with the intake of antioxidant vitamins, minerals, dietary fiber, and ω−3 fatty acids [28]. Our study emphasized that traditional Japanese food intake, like the Mediterranean diet [13], has a preventive effect on cardiometabolic risks.

We observed a significantly positive association of the juice/milk dietary pattern with high TC and high LDL-C concentrations in the main and sensitivity analyses. We also observed a significantly positive association of the juice/milk dietary pattern with the risk of impaired blood glucose control in 2014 (Sensitivity 2) and 2015 (Model 2 in Table 5). This might be because of the higher intake of dairy products and sugar-based juices, like the high-dairy [24] and bread [42] dietary patterns. The juice/milk dietary pattern in this study can be considered the reverse of the traditional Japanese staple food pattern [42]. Considering the diverse association in the main and sensitivity analyses and between 2014 and 2015, for both vegetable and juice/milk dietary patterns, further studies on associations with diabetes risk are required.

For the meat dietary pattern, our results were consistent in some ways with other studies on inverse associations with high triglycerides and low HDL-C concentrations in a Japanese population [15], inverse associations with high TC and high LDL-C concentrations in a Korean population [43], and increasing HDL levels with increasing meat intake in a young Brazilian population [41]. The Ohsaki Cohort Study developed the Japanese Diet Index with nine food items, including those of the vegetable dietary pattern in our study and green tea, beef, pork, and coffee [44]. The low saturated fat (meat) and high ω-3 polyunsaturated fat (fish) in the Japanese diet contribute to the low prevalence of hypercholesterolemia [45]. In this study, the meat dietary pattern that was protective against the risk of low HDL-C concentration in the 2015 dataset, but not in the 2014 dataset, supports this notion.

One of the strengths of this study was that we computed the accumulative means of dietary pattern scores arising from repeated FFQ surveys and could attenuate misclassifications to more accurately reflect dietary stability [39]. On the basis of the large sample and careful handling of missing data from FFQs [46], although small in this study, the reliability of the three patterns in our study was higher compared to other similar studies [15]. Second, we estimated the associations of health checkup measurements in 2014 and 2015 after dietary surveys were conducted between 2011 and 2013. In such a longitudinal way, the associations measured were more robust than simple cross-sectional surveys. Third, the same associations were observed for both the 2014 and the 2015 dataset, as well in as the sensitivity analysis, which enhanced the study results. In addition, our results were more stable in 2015 than in 2014, which might also indicate that the long-term effects of dietary patterns are more prominent.

This study had a few limitations. Firstly, the FHMS response rates remained at ~27% [47], in which 45% of participants had changed living places. Therefore, the representativeness of our results might not be generalizable to the entire prefecture or the country’s population. Second, we could not compute the food amount or nutrients to derive dietary patterns. Without nutrient intake, it is difficult to evaluate the nutritional status, which is straightforward in the elucidation of underlying biological mechanisms [17,48]. Also, no hormonal information such as insulin and leptin was available for further analysis. Finally, food consumption was self-reported and the dietary report generally underreported [21]. A total of 19 food items might not be sufficient for examining correlations between specific foods, although the FFQs had the same food groups as other studies [49,50].

In summary, three dietary patterns were identified using three waves of food intake surveys between 2011 and 2013. The vegetable dietary pattern might be inversely associated with cardiometabolic risks including overweight, hypertension, and dyslipidemia, in a longitudinal way, while the juice/milk dietary pattern might be positively associated with the risk of impaired blood glucose control and dyslipidemia. The meat dietary pattern might also be protective against dyslipidemia risk. The sensitivity analysis provided confirmed significant results, prominently for the association between the vegetable pattern and dyslipidemia risk. Considering the long-term effects of diets on cardiometabolic risks, our study may suggest that the continuous promotion of the vegetable dietary pattern rich in traditional Japanese foods is necessary to reduce cardiometabolic risks, particularly for dyslipidemia.

## Figures and Tables

**Table 1:** Characteristics of participants (2011–2013) and their health checkup results (2014–2015) stratified by the cumulative means of vegetable dietary pattern scores.

	2014 (*n* = 15,409)						2015 (*n* = 14,999)					
	All	Q1	Q2	Q3	Q4	*p*-Value	All	Q1	Q2	Q3	Q4	*p*-Value
Age (years)	62 (52, 69)	53 (37, 62)	61 (49, 67)	64 (57, 70)	68 (62, 73)	<0.001	62 (52, 69)	53 (38, 62)	61 (49, 67)	64 (57, 70)	68 (62, 73)	<0.001
Women (%)	60.6	57.8	57.2	61.0	66.4	<0.001	61.1	56.6	59.5	61.8	66.6	<0.001
Education ≥ vocational university (%)	24.4	27.5	25.5	21.7	22.8	<0.001	24.5	27.4	26.2	21.4	23.1	<0.001
Current smoker (%)	14.8	23.8	17.0	11.0	7.5	<0.001	14.9	23.7	16.7	11.9	7.4	<0.001
Current alcohol drinking (%)	45.1	47.2	48.2	44.6	40.5	<0.001	45.9	49.2	47.3	45.4	41.5	<0.001
Physical activity ≥ 2 times/week (%)	41.3	24	35.7	46.6	58.9	<0.001	41.0	24.2	35.5	45.7	58.5	<0.001
Distress scale ≥ 13 (%)	12.3	14.4	11.8	11.3	11.6	<0.001	12.3	15.0	11.5	11.3	11.3	<0.001
Live at shelter/temporary/rental house (%)	44.6	52.7	45.9	41.1	38.6	<0.001	45.2	52.9	45.9	43.0	38.9	<0.001
BMI (kg/m^2^)	23 (21, 26)	23 (21, 26)	23 (21, 26)	23 (21, 26)	23 (21, 25)	0.354	23 (21, 26)	23 (21, 26)	23 (21, 26)	23 (21, 26)	23 (21, 25)	0.007
BMI ≥ 25 kg/m^2^ (%)	30.1	31.3	30.6	30.0	28.6	0.071	30.1	31.6	31.4	30.2	27.2	<0.001
Hypertension (%)	44.8	33.4	43	49.3	53.6	<0.001	45.9	35.2	44.7	50.1	53.4	<0.001
SBP (mmHg)	126 (114, 136)	122 (110, 132)	124 (114, 134)	128 (116, 136)	128 (118, 138)	<0.001	126 (116, 136)	122 (112, 132)	126 (114, 134)	128 (118, 136)	130 (120, 138)	<0.001
DBP (mmHg)	74 (68, 80)	74 (66, 80)	74 (68, 80)	74 (68, 80)	74 (68, 80)	0.036	74 (68, 80)	74 (68, 82)	74 (68, 82)	74 (68, 80)	74 (68, 80)	0.929
Fast blood glucose (mg/dL)	96 (89, 104)	93 (87, 101)	95 (89, 103)	96 (90, 105)	97 (91, 106)	<0.001	96 (90, 105)	94 (88, 103)	96 (90, 105)	97 (90, 106)	97 (91, 107)	<0.001
Fast blood glucose ≥ 126 mg/dL (%)	6.1	5.2	5.7	6.8	6.6	0.01	6.1	5.2	6.1	6.4	6.8	0.014
HbA1c1 (%)	5.5 (5.3, 5.8)	5.4 (5.2, 5.7)	5.5 (5.3, 5.8)	5.5 (5.3, 5.8)	5.6 (5.3, 5.8)	<0.001	5.5 (5.3, 5.8)	5.4 (5.2, 5.7)	5.5 (5.3, 5.8)	5.6 (5.3, 5.8)	5.6 (5.4, 5.9)	<0.001
HbA1c1 ≥ 6.5% (%)	6.6	5.6	6.1	7.3	7.4	0.002	7.3	6.2	7.2	7.6	8	0.024
Total cholesterol (mg/dL)	229 (204, 258)	231 (203, 262)	230 (204, 259)	230 (204, 258)	227 (203, 254)	<0.001	230 (204, 258)	229 (203, 261)	231 (206, 259)	229 (204, 257)	229 (204, 254)	0.010
Total cholesterol ≥ 220 mg/dL (%)	59.2	60	59.8	59.4	57.8	0.181	59.5	58.6	60.9	59.9	58.7	0.143
LDL-C (mg/dL)	120 (101, 141)	121 (100, 144)	121 (101, 142)	120 (100, 141)	120 (101, 139)	0.005	120 (101, 141)	121 (100, 143)	122 (102, 142)	120 (101, 141)	119 (101, 139)	0.007
LDL-C ≥ 140 mg/dL (%)	27	29.2	27.9	26.3	24.7	<0.001	26.8	28.6	27.6	26.1	25.0	0.002
HDL-C (mg/dL)	59 (50, 70)	59 (50, 70)	59 (50, 70)	60 (50, 71)	60 (51, 70)	0.318	60 (50, 71)	60 (50, 71)	59 (50, 70)	60 (50, 71)	60 (51, 71)	0.098
HDL-C < 40 mg/dL (%)	5.3	5.8	5.3	5.3	4.9	0.370	5.4	6.2	5.3	5.4	4.5	0.011
Triglycerides (mg/dL)	93 (67, 131)	93 (64, 136)	92 (66, 132)	94 (68, 132)	92 (68, 126)	0.586	92 (66, 131)	91 (63, 135)	94 (67, 133)	92 (67, 130)	91 (68, 125)	0.216
Triglycerides ≥ 150 mg/dL (%)	18.1	19.9	18.6	18.2	15.6	<0.001	17.8	20.1	18.2	17.7	15.1	<0.001
Metabolic syndrome (%)	9.2	9.2	10.5	9.7	7.5	0.067	9.6	10.3	10.9	9.7	7.7	0.096
Vegetable dietary pattern score	0.01 (−0.66, 0.68)	−1.11 (−1.46, −0.86)	−0.31 (−0.48, −0.15)	0.34 (0.18, 0.50)	1.12 (0.88, 1.43)	<0.001	0 (−0.67, 0.67)	−1.12 (−1.46, −0.87)	−0.32 (−0.48, −0.16)	0.33 (0.17, 0.49)	1.11 (0.87, 1.43)	<0.001

Results expressed as a median (25th and 75th percentile) or a percentage. *p*-Values resulting from nonparametric ANOVA comparing the median or chi-square test comparing proportions across the four quartiles. BMI, body mass index; SBP, systolic blood pressure; DBP, diastolic blood pressure; LDL-C, low-density lipoprotein cholesterol; HDL-C, high-density lipoprotein cholesterol; ANOVA, analysis of variance.

**Table 2:** Characteristics of participants (2011–2013) and their health checkup results (2014–2015) stratified by the cumulative means of juice/milk dietary pattern scores.

	2014 (*n* = 15,409)						2015 (*n* = 14,999)					
	All	Q1	Q2	Q3	Q4	*p*-Value	All	Q1	Q2	Q3	Q4	*p*-Value
Age (years)	62 (52, 69)	61 (48, 68)	62 (51, 69)	62 (51, 70)	64 (55, 71)	<0.001	62 (52, 69)	61 (50, 67)	62 (51, 69)	62 (52, 69)	63 (55, 70)	<0.001
Women (%)	39.4	48.8	59.2	65.4	69.0	<0.001	61.1	50.1	60.4	65.0	69.1	<0.001
Education ≥ vocational university (%)	24.4	20.9	22.6	26.5	27.5	<0.001	24.5	21.2	23.1	25.8	27.9	<0.001
Current smoker (%)	14.8	22.5	15.0	11.9	9.9	<0.001	14.9	22.4	14.7	12.2	10.5	<0.001
Current alcohol drinking (%)	45.1	53.7	45.3	43.3	38.2	<0.001	45.9	54.7	45.5	44.0	39.3	<0.001
Physical activity ≥ 2 times/week (%)	41.3	33.4	37.3	43.1	51.4	<0.001	41.0	32.2	37.3	43.7	50.7	<0.001
Distress scale ≥ 13 (%)	12.3	11.2	12.0	12.3	13.6	0.013	12.3	11.8	12.6	11.9	12.8	0.428
Live at shelter/temporary/rental house (%)	44.6	42.9	44.8	44.8	45.8	0.096	45.2	44.3	45.1	45.0	46.3	0.187
BMI (kg/m^2^)	23 (21, 26)	23 (21, 26)	23 (21, 26)	23 (21, 25)	23 (21, 26)	0.017	23 (21, 26)	23 (21, 26)	23 (21, 26)	23 (21, 25)	23 (21, 26)	0.002
BMI ≥ 25 kg/m^2^ (%)	30.1	31.2	31.1	28.8	29.4	0.052	30.1	32.0	30.4	28.6	29.6	0.012
Hypertension (%)	44.8	44.5	44.7	44.2	45.8	0.626	45.9	48.0	45.9	44.4	45.1	0.012
SBP (mmHg)	126 (114, 136)	126 (114, 136)	126 (114, 136)	125 (114, 135)	126 (115, 135)	0.055	126 (116, 136)	126 (116, 136)	126 (116, 136)	126 (114, 136)	126 (116, 136)	0.169
DBP (mmHg)	74 (68, 80)	74 (68, 82)	74 (68, 80)	74 (68, 80)	74 (68, 80)	0.004	74 (68, 80)	76 (68, 82)	74 (68, 81)	74 (68, 80)	74 (68, 80)	<0.001
Fast blood glucose (mg/dL)	96 (89, 104)	96 (89, 104)	96 (89, 104)	95 (89, 104)	96 (89, 104)	0.575	96 (90, 105)	96 (90, 106)	96 (90, 104)	96 (89, 105)	96 (90, 105)	0.145
Fast blood glucose ≥ 126 mg/dL (%)	6.1	6.4	6.0	5.9	6.1	0.696	6.1	6.1	6.1	6.2	6.0	0.960
HbA1c1 (%)	5.5 (5.3, 5.8)	5.5 (5.3, 5.7)	5.5 (5.3, 5.8)	5.5 (5.3, 5.8)	5.5 (5.3, 5.8)	<0.001	5.5 (5.3, 5.8)	5.5 (5.3, 5.8)	5.5 (5.3, 5.8)	5.5 (5.3, 5.8)	5.6 (5.3, 5.9)	<0.001
HbA1c1 ≥ 6.5% (%)	6.6	6.3	6.6	6.3	7.2	0.364	7.3	6.7	7.0	7.3	8.0	0.166
Total cholesterol (mg/dL)	229 (204, 258)	227 (201, 257)	228 (204, 258)	229 (204, 257)	232 (207, 260)	<0.001	230 (204, 258)	227 (201, 257)	229 (204, 257)	230 (205, 258)	232 (207, 259)	<0.001
Total cholesterol ≥ 220 mg/dL (%)	59.2	57.0	58.6	59.0	62.3	<0.001	59.5	57.2	58.5	59.7	62.6	<0.001
LDL-C (mg/dL)	120 (101, 141)	118 (98, 139)	120 (100, 142)	121 (101, 142)	122 (103, 143)	<0.001	120 (101, 141)	118 (99, 139)	120 (100, 141)	121 (101, 142)	122 (103, 143)	<0.001
LDL-C ≥ 140 mg/dL (%)	27	25.0	27.7	27.7	27.8	0.012	26.8	24.9	26.2	27.4	28.7	0.002
HDL-C (mg/dL)	59 (50, 70)	58 (49, 70)	59 (50, 70)	60 (51, 71)	60 (51, 71)	<0.001	60 (50, 71)	58 (49, 70)	59 (50, 70)	60 (51, 71)	61 (51, 72)	<0.001
HDL-C < 40 mg/dL (%)	5.3	6.4	5.3	5.1	4.4	0.001	5.4	6.2	5.5	5.5	4.3	0.005
Triglycerides (mg/dL)	93 (67, 131)	93 (66, 135)	93 (67, 131)	90 (65, 128)	94 (68, 131)	0.002	92 (66, 131)	93 (67, 134)	92 (67, 131)	90 (64, 130)	93 (67, 129)	0.007
Triglycerides ≥ 150 mg/dL (%)	18.1	20.0	17.8	16.4	18.1	0.001	17.8	19.0	17.9	17.1	17.0	0.078
Metabolic syndrome (%)	9.2	9.9	9.4	9.1	8.6	0.295	9.6	10.6	9.4	9.3	9.3	0.342
Juice/milk dietary pattern score	−0.15 (−0.64, 0.47)	−0.94 (−1.16, −0.78)	−0.4 (−0.51, −0.28)	0.13 (−0.02, 0.28)	1.02 (0.69, 1.55)	<0.001	−0.14 (−0.65, 0.46)	−0.95 (−1.17, −0.78)	−0.4 (−0.52, −0.28)	0.13 (−0.01, 0.28)	1.02 (0.69, 1.57)	<0.001

Results expressed as a median (25th and 75th percentile) or a percentage. *p*-values resulting from nonparametric ANOVA comparing median or chi-square test comparing proportions across the four quartiles. BMI, body mass index; SBP, systolic blood pressure; DBP, diastolic blood pressure; LDL-C, low-density lipoprotein cholesterol; HDL-C, high-density lipoprotein cholesterol; ANOVA, analysis of variance.

**Table 3:** Characteristics of participants (2011–2013) and their health checkup results (2014–2015) stratified by the cumulative means of meat dietary pattern scores.

	2014 (*n* = 15,402)						2015 (*n* = 14,998)					
	All	Q1	Q2	Q3	Q4	*p*-Value	All	Q1	Q2	Q3	Q4	*p*-Value
Age (years)	62 (52, 69)	65 (60, 72)	63 (55, 70)	61 (48, 68)	57 (40, 66)	<0.001	62 (52, 69)	65 (59, 71)	62 (55, 69)	61 (49, 68)	58 (40, 67)	<0.001
Women (%)	60.6	57.1	57.6	63.1	64.6	<0.001	61.1	56.6	58.2	64.2	65.6	<0.001
Education ≥ vocational university (%)	24.4	18.0	22.9	26.2	30.3	<0.001	24.5	18.0	23.0	26.5	30.5	<0.001
Current smoker (%)	14.8	11.5	14.6	16.5	16.6	<0.001	14.9	11.8	15.2	16.4	16.3	<0.001
Current alcohol drinking (%)	45.1	43.8	47.0	45.2	44.4	0.004	45.9	44.8	48.7	45.8	44.2	0.001
Physical activity ≥ 2 times/week (%)	41.3	47.8	41.2	38.4	37.7	<0.001	41.0	46.9	41.7	38.4	36.9	<0.001
Distress scale ≥ 13 (%)	12.3	12.5	12.5	11.9	12.3	0.810	12.3	12.8	11.8	12.1	12.5	0.532
Live at shelter/temporary/rental house (%)	44.6	43.8	45.4	44.6	44.6	0.66	45.2	44.3	45.2	45.4	45.7	0.743
BMI (kg/m^2^)	23 (21, 26)	23 (21, 26)	23 (21, 26)	23 (21, 25)	23 (21, 26)	<0.001	23 (21, 26)	24 (21, 26)	23 (21, 26)	23 (21, 25)	23 (21, 26)	<0.001
BMI ≥ 25 kg/m^2^ (%)	30.1	31.6	32.1	27.8	29.0	<0.001	30.1	31.4	31.0	28.6	29.5	0.024
Hypertension (%)	44.8	53.5	47.4	41.5	36.8	<0.001	45.9	54.7	48.1	42.7	37.9	<0.001
SBP (mmHg)	126 (114, 136)	128 (118, 138)	126 (116, 136)	124 (112, 134)	122 (111, 134)	<0.001	126 (116, 136)	128 (120, 138)	128 (116, 136)	126 (114, 134)	124 (112, 134)	<0.001
DBP (mmHg)	74 (68, 80)	75 (68, 81)	74 (68, 82)	74 (66, 80)	73 (66, 80)	<0.001	74 (68, 80)	76 (69, 82)	74 (68, 82)	74 (68, 80)	74 (67, 80)	<0.001
Fast blood glucose (mg/dL)	96 (89, 104)	97 (91, 106)	96 (90, 104)	95 (89, 103)	94 (88, 102)	<0.001	96 (90, 105)	98 (91, 107)	96 (90, 105)	95 (89, 104)	94 (88, 103)	<0.001
Fast blood glucose ≥ 126 mg/dL (%)	6.1	7.1	6.1	6.0	5.1	0.007	6.1	7.4	5.8	6.1	5.2	<0.001
HbA1c1 (%)	5.5 (5.3, 5.8)	5.5 (5.3, 5.8)	5.5 (5.3, 5.8)	5.5 (5.3, 5.8)	5.5 (5.3, 5.7)	<0.001	5.5 (5.3, 5.8)	5.6 (5.4, 5.9)	5.5 (5.3, 5.8)	5.5 (5.3, 5.8)	5.5 (5.3, 5.8)	<0.001
HbA1c1 ≥ 6.5% (%)	6.6	7.8	6.8	6.3	5.5	0.001	7.3	8.6	7.0	7.3	6.2	<0.001
Total cholesterol (mg/dL)	229 (204, 258)	229 (204, 257)	230 (204, 259)	231 (205, 259)	227 (201, 257)	0.002	230 (204, 258)	229 (205, 257)	232 (205, 259)	231 (205, 258)	227 (201, 257)	<0.001
Total cholesterol ≥ 220 mg/dL (%)	59.2	59.9	60.1	60.4	56.6	0.001	59.5	59.2	61.7	60.3	57.0	<0.001
LDL-C (mg/dL)	120 (101, 141)	120 (101, 140)	121 (101, 142)	121 (102, 142)	119 (99, 141)	0.020	120 (101, 141)	119 (101, 140)	120 (101, 141)	121 (102, 142)	120 (99, 141)	0.120
LDL-C ≥ 140 mg/dL (%)	27.0	25.9	27.2	28.3	26.7	0.139	26.8	26.0	26.5	27.8	26.9	0.323
HDL-C (mg/dL)	59 (50, 70)	58 (49, 69)	59 (50, 70)	60 (51, 71)	61 (51, 71)	<0.001	60 (50, 71)	58 (49, 69)	60 (50, 70)	60 (51, 71)	61 (51, 72)	<0.001
HDL-C < 40 mg/dL (%)	5.3	6.3	5.7	4.6	4.6	0.001	5.4	6.6	5.5	5.1	4.2	<0.001
Triglycerides (mg/dL)	93 (67, 131)	97 (71, 134)	94 (68, 133)	91 (65, 129)	87 (63, 127)	<0.001	92 (66, 131)	97 (72, 135)	94 (68, 134)	91 (65, 130)	87 (62, 124)	<0.001
Triglycerides ≥ 150 mg/dL (%)	18.1	18.6	19.0	17.5	17.2	0.107	17.8	19.1	19.0	17.2	15.8	<0.001
Metabolic syndrome (%)	9.2	10.2	10.7	8.8	7.3	<0.001	9.6	10.7	10.3	9.3	8.4	<0.001
Juice/milk dietary pattern score	−0.16 (−0.60, 0.46)	−0.87 (−1.09, −0.72)	−0.39 (−0.49, −0.28)	0.11 (−0.03, 0.27)	0.99 (0.68, 1.48)	<0.001	−0.16 (−0.61, 0.45)	−0.88 (−1.10, −0.73)	−0.38 (−0.49, −0.27)	0.12 (−0.03, 0.27)	1.00 (0.69, 1.48)	<0.001

Results expressed as a median (25th and 75th percentile) or a percentage. *p*-Values resulting from nonparametric ANOVA comparing median or chi-square test comparing proportions across the four quartiles. BMI, body mass index; SBP, systolic blood pressure; DBP, diastolic blood pressure; LDL-C, low-density lipoprotein cholesterol; HDL-C, high-density lipoprotein cholesterol; ANOVA, analysis of variance.

**Table 4:** Prevalence ratios (PRs, 95% CIs) for cardiometabolic factors in 2014 by quartiles of accumulative means of dietary pattern scores during 2011–2013, FHMS.

	BMI ≥ 25 (kg/m^2^)	Hypertension	Fasting Blood Glucose ≥ 126 (mg/dL)	HbA1c1 ≥ 6.5%	TC ≥ 220 (mg/dL)	LDL-C ≥ 140 (mg/dL)	HDL-C < 40 (mg/dL)	Triglycerides ≥ 150 (mg/dL)	MetS
**Vegetable**									
Model 1	1.00 (ref.)	1.00 (ref.)	1.00 (ref.)	1.00 (ref.)	1.00 (ref.)	1.00 (ref.)	1.00 (ref.)	1.00 (ref.)	1.00 (ref.)
	**0.98 (0.97–1.00)**	1.00 (0.98–1.01)	0.99 (0.98–1.00)	0.99 (0.98–10)	0.99 (0.97–1.00)	0.99 (0.97–1.00)	0.99 (0.98–1.00)	**0.98 (0.97–1.00)**	1.00 (0.98–1.01)
	**0.97 (0.96–0.99)**	0.99 (0.97–1.00)	1.00 (0.99–1.01)	1.00 (0.99–1.01)	**0.97 (0.96–0.99)**	**0.97 (0.96–0.99)**	**0.99 (0.98–1.00)**	**0.98 (0.97–1.00)**	0.99 (0.97–1.00)
	**0.96 (0.94–0.98)**	**0.98 (0.96–0.99)**	0.99 (0.98–1.01)	1.00 (0.99–1.01)	**0.96 (0.94–0.97)**	**0.96 (0.94–0.97)**	**0.99 (0.98–1.00)**	**0.96 (0.95–0.98)**	**0.97 (0.95–0.99)**
*p* trend	**<0.0001**	**0.005**	0.525	0.993	**<0.0001**	**<0.0001**	**0.018**	**<0.0001**	**0.0003**
Model 2	1.00 (ref.)	1.00 (ref.)	1.00 (ref.)	1.00 (ref.)	1.00 (ref.)	1.00 (ref.)	1.00 (ref.)	1.00 (ref.)	1.00 (ref.)
	0.99 (0.97–1.00)	0.99 (0.98–1.01)	0.99 (0.98–1.01)	1.00 (0.99–1.01)	0.99 (0.97–1.00)	0.99 (0.97–1.01)	0.99 (0.98–1.00)	0.99 (0.97–1.00)	1.00 (0.98–1.01)
	**0.98 (0.97–1.00)**	0.99 (0.97–1.00)	1.00 (0.99–1.01)	1.00 (0.99–1.01)	**0.98 (0.96–0.99)**	**0.98 (0.96–0.99)**	0.99 (0.98–1.00)	0.99 (0.98–1.01)	0.99 (0.97–1.00)
	**0.97 (0.96–0.99)**	**0.98 (0.97–1.00)**	1.00 (0.98–1.01)	1.00 (0.99–1.01)	**0.96 (0.95–0.97)**	**0.96 (0.95–0.98)**	0.99 (0.98–1.00)	**0.97 (0.96–0.99)**	**0.98 (0.96–0.99)**
*p* trend	**0.002**	**0.01**	0.609	0.964	**<0.0001**	**<0.0001**	0.182	**0.003**	**0.004**
Sensitivity 1	1.00 (ref.)	1.00 (ref.)	1.00 (ref.)	1.00 (ref.)	1.00 (ref.)	1.00 (ref.)	1.00 (ref.)	1.00 (ref.)	1.00 (ref.)
	0.99 (0.97–1.01)	0.99 (0.97–1.01)	0.99 (0.98–1.01)	1.00 (0.98–1.01)	**0.98 (0.97–1.00)**	**0.98 (0.96–1.00)**	0.99 (0.98–1.01)	0.99 (0.97–1.01)	0.99 (0.97–1.01)
	0.99 (0.97–1.01)	**0.97 (0.96–0.99)**	0.99 (0.98–1.01)	0.99 (0.98–1.01)	**0.97 (0.95–0.98)**	**0.97 (0.95–0.99)**	1.00 (0.98–1.01)	0.98 (0.96–1.00)	**0.98 (0.96–1.00)**
	**0.97 (0.95–1.00)**	**0.98 (0.96–1.00)**	0.99 (0.98–1.01)	1.00 (0.98–1.01)	**0.96 (0.94–0.98)**	**0.96 (0.94–0.99)**	0.99 (0.98–1.00)	**0.97 (0.95–0.99)**	**0.96 (0.94–0.98)**
*p* trend	**0.028**	**0.008**	0.333	0.664	**<0.0001**	**0.002**	0.159	**0.007**	**0.0001**
Sensitivity 2	1.00 (ref.)	1.00 (ref.)	1.00 (ref.)	1.00 (ref.)	1.00 (ref.)	1.00 (ref.)	1.00 (ref.)	1.00 (ref.)	1.00 (ref.)
	1.00 (0.98–1.03)	0.99 (0.98–1.01)	1.00 (0.99–1.00)	1.00 (0.99–1.00)	1.00 (0.98–1.01)	0.99 (0.97–1.01)	1.00 (0.98–1.01)	1.00 (0.98–1.02)	1.01 (0.99–1.02)
	0.99 (0.96–1.01)	0.98 (0.97–1.00)	1.00 (0.99–1.01)	1.00 (0.99–1.01)	**0.98 (0.97–1.00)**	0.98 (0.96–1.00)	0.99 (0.98–1.01)	1.00 (0.98–1.01)	0.99 (0.98–1.01)
	0.97 (0.95–1.00)	1.00 (0.98–1.02)	0.99 (0.98–1.00)	1.00 (0.99–1.00)	**0.96 (0.95–0.98)**	**0.96 (0.94–0.98)**	0.99 (0.98–1.00)	**0.98 (0.96–1.00)**	0.99 (0.98–1.01)
*p* trend	**0.027**	0.909	0.068	0.483	**<0.0001**	**<0.0001**	0.062	**0.037**	0.224
**Juice/Milk**									
Model 1	1.00 (ref.)	1.00 (ref.)	1.00 (ref.)	1.00 (ref.)	1.00 (ref.)	1.00 (ref.)	1.00 (ref.)	1.00 (ref.)	1.00 (ref.)
	1.00 (0.99–1.02)	0.99 (0.98–1.01)	1.00 (0.99–1.01)	1.01 (1.00–1.02)	1.01 (0.99–1.02)	**1.02 (1.00–1.03)**	1.00 (0.99–1.01)	0.99 (0.98–1.01)	1.00 (0.99–1.02)
	0.99 (0.98–1.01)	0.99 (0.98–1.00)	1.00 (0.99–1.01)	1.00 (0.99–1.01)	1.01 (0.99–1.02)	**1.02 (1.00–1.03)**	1.00 (0.99–1.01)	**0.98 (0.97–1.00)**	1.00 (0.99–1.02)
	1.00 (0.98–1.01)	**0.98 (0.97–1.00)**	1.00 (0.99–1.01)	1.01 (1.00–1.02)	**1.03 (1.01–1.04)**	**1.02 (1.00–1.03)**	0.99 (0.98–1.00)	1.00 (0.99–1.02)	1.00 (0.99–1.02)
*p* trend	0.34	**0.017**	0.715	0.062	**0.0004**	0.062	0.151	0.805	0.686
Model 2	1.00 (ref.)	1.00 (ref.)	1.00 (ref.)	1.00 (ref.)	1.00 (ref.)	1.00 (ref.)	1.00 (ref.)	1.00 (ref.)	1.00 (ref.)
	1.01 (0.99–1.02)	1.00 (0.98–1.01)	1.00 (0.99–1.01)	1.01 (0.99–1.02)	1.01 (0.99–1.02)	**1.02 (1.00–1.04)**	1.00 (0.99–1.01)	1.00 (0.98–1.01)	1.00 (0.99–1.02)
	1.00 (0.98–1.01)	0.99 (0.98–1.01)	1.00 (0.99–1.01)	1.00 (0.99–1.01)	1.01 (1.00–1.02)	**1.02 (1.00–1.04)**	1.00 (0.99–1.01)	0.99 (0.98–1.01)	1.01 (0.99–1.02)
	1.00 (0.98–1.02)	0.99 (0.98–1.00)	1.00 (0.99–1.01)	1.01 (1.00–1.02)	**1.03 (1.01–1.04)**	**1.02 (1.01–1.04)**	0.99 (0.98–1.00)	1.01 (1.00–1.03)	1.01 (0.99–1.02)
*p* trend	0.837	0.128	0.668	0.172	**<0.0001**	**0.018**	0.227	0.125	0.462
Sensitivity 1	1.00 (ref.)	1.00 (ref.)	1.00 (ref.)	1.00 (ref.)	1.00 (ref.)	1.00 (ref.)	1.00 (ref.)	1.00 (ref.)	1.00 (ref.)
	1.01 (0.99–1.03)	1.00 (0.98–1.02)	1.00 (0.98–1.01)	1.00 (0.99–1.02)	1.00 (0.98–1.02)	1.01 (0.99–1.03)	0.99 (0.98–1.01)	0.99 (0.97–1.01)	1.01 (0.99–1.03)
	0.99 (0.97–1.01)	0.99 (0.98–1.01)	1.00 (0.99–1.02)	1.00 (0.99–1.01)	1.01 (0.99–1.03)	1.02 (1.00–1.04)	0.99 (0.98–1.00)	**0.98 (0.96–1.00)**	1.01 (0.99–1.03)
	1.00 (0.98–1.02)	0.99 (0.97–1.01)	1.00 (0.98–1.01)	1.00 (0.99–1.02)	**1.02 (1.00–1.04)**	1.01 (0.99–1.04)	0.99 (0.98–1.01)	1.00 (0.98–1.02)	1.00 (0.98–1.02)
*p* trend	0.481	0.313	0.864	0.71	**0.008**	0.192	0.332	0.769	0.789
Sensitivity 2	1.00 (ref.)	1.00 (ref.)	1.00 (ref.)	1.00 (ref.)	1.00 (ref.)	1.00 (ref.)	1.00 (ref.)	1.00 (ref.)	1.00 (ref.)
	0.99 (0.97–1.01)	1.01 (0.99–1.03)	1.00 (0.99–1.00)	1.00 (0.99–1.00)	1.01 (1.00–1.03)	**1.02 (1.01–1.04)**	1.00 (0.99–1.01)	1.00 (0.98–1.01)	**0.98 (0.97–1.00)**
	1.01 (0.99–1.03)	1.01 (0.99–1.02)	1.00 (0.99–1.01)	1.00 (1.00–1.01)	**1.02 (1.00–1.04)**	**1.03 (1.01–1.05)**	1.00 (0.99–1.01)	0.99 (0.98–1.01)	0.99 (0.97–1.00)
	1.00 (0.98–1.03)	1.00 (0.98–1.02)	1.00 (0.99–1.01)	**1.01 (1.00–1.02)**	**1.04 (1.02–1.05)**	**1.04 (1.02–1.06)**	0.99 (0.98–1.01)	1.01 (0.99–1.03)	0.99 (0.97–1.00)
*p* trend	0.494	0.854	0.574	**0.008**	**<0.0001**	**<0.0001**	0.313	0.351	0.187
**Meat**									
Model 1	1.00 (ref.)	1.00 (ref.)	1.00 (ref.)	1.00 (ref.)	1.00 (ref.)	1.00 (ref.)	1.00 (ref.)	1.00 (ref.)	1.00 (ref.)
	1.01 (0.99–1.02)	1.00 (0.99–1.01)	1.00 (0.99–1.01)	1.00 (0.99–1.01)	1.01 (0.99–1.02)	1.01 (0.99–1.03)	1.00 (0.99–1.01)	1.01 (0.99–1.02)	1.01 (0.99–1.03)
	0.98 (0.97–1.00)	0.99 (0.98–1.00)	1.00 (0.99–1.01)	1.00 (0.99–1.01)	1.01 (1.00–1.03)	1.02 (1.00–1.03)	0.99 (0.98–1.00)	1.00 (0.98–1.01)	1.00 (0.98–1.01)
	1.00 (0.98–1.01)	0.99 (0.98–1.01)	1.00 (0.99–1.01)	1.00 (0.98–1.01)	0.99 (0.98–1.01)	1.00 (0.99–1.02)	0.99 (0.98–1.00)	1.00 (0.98–1.01)	0.99 (0.98–1.01)
*p* trend	0.246	0.143	0.752	0.453	0.695	0.695	0.16	0.578	0.21
Model 2	1.00 (ref.)	1.00 (ref.)	1.00 (ref.)	1.00 (ref.)	1.00 (ref.)	1.00 (ref.)	1.00 (ref.)	1.00 (ref.)	1.00 (ref.)
	1.01 (0.99–1.03)	1.00 (0.99–1.01)	1.00 (0.99–1.01)	1.00 (0.99–1.01)	1.00 (0.99–1.02)	1.01 (0.99–1.02)	1.00 (0.99–1.01)	1.00 (0.99–1.02)	1.01 (0.99–1.03)
	**0.99 (0.97–1.00)**	0.99 (0.98–1.01)	1.00 (0.99–1.01)	1.00 (0.99–1.01)	1.01 (1.00–1.02)	**1.02 (1.00–1.03)**	0.99 (0.98–1.00)	1.00 (0.98–1.01)	1.00 (0.98–1.02)
	1.00 (0.98–1.02)	0.99 (0.98–1.01)	1.00 (0.99–1.01)	1.00 (0.98–1.01)	0.99 (0.98–1.00)	1.00 (0.99–1.02)	0.99 (0.98–1.00)	1.00 (0.98–1.01)	1.00 (0.98–1.01)
*p* trend	0.493	0.257	0.715	0.452	0.14	0.719	0.143	0.535	0.334
Sensitivity 1	1.00 (ref.)	1.00 (ref.)	1.00 (ref.)	1.00 (ref.)	1.00 (ref.)	1.00 (ref.)	1.00 (ref.)	1.00 (ref.)	1.00 (ref.)
	1.01 (0.99–1.03)	1.00 (0.98–1.02)	0.99 (0.98–1.00)	0.99 (0.98–1.01)	1.01 (1.00–1.03)	1.01 (0.99–1.03)	1.00 (0.99–1.01)	1.00 (0.99–1.02)	1.01 (0.99–1.03)
	0.98 (0.96–1.00)	0.99 (0.97–1.01)	1.00 (0.99–1.02)	1.00 (0.98–1.01)	1.01 (0.99–1.02)	1.01 (0.99–1.03)	0.99 (0.98–1.01)	0.98 (0.97–1.00)	0.99 (0.97–1.01)
	1.00 (0.98–1.02)	1.00 (0.98–1.02)	0.99 (0.98–1.01)	0.99 (0.98–1.01)	0.99 (0.97–1.01)	1.00 (0.98–1.02)	0.99 (0.98–1.01)	0.99 (0.97–1.01)	1.00 (0.98–1.02)
*p* trend	0.591	0.739	0.719	0.275	0.147	0.903	0.257	0.11	0.929
Sensitivity 2	1.00 (ref.)	1.00 (ref.)	1.00 (ref.)	1.00 (ref.)	1.00 (ref.)	1.00 (ref.)	1.00 (ref.)	1.00 (ref.)	1.00 (ref.)
	1.01 (0.99–1.03)	1.00 (0.98–1.02)	1.00 (0.99–1.01)	1.00 (0.99–1.01)	1.00 (0.98–1.02)	1.00 (0.98–1.02)	1.00 (0.99–1.01)	1.00 (0.99–1.02)	0.98 (0.97–1.00)
	0.98 (0.96–1.00)	1.00 (0.98–1.01)	1.00 (0.99–1.01)	1.00 (0.99–1.01)	1.01 (0.99–1.02)	1.01 (1.00–1.03)	0.99 (0.98–1.00)	1.00 (0.98–1.02)	0.99 (0.98–1.01)
	1.00 (0.98–1.03)	1.01 (0.99–1.02)	1.00 (0.99–1.01)	1.00 (0.99–1.01)	**0.98 (0.97–1.00)**	1.00 (0.98–1.01)	0.99 (0.98–1.00)	1.00 (0.98–1.02)	1.00 (0.98–1.01)
*p* trend	0.66	0.437	0.956	0.732	**0.029**	0.606	0.179	0.848	0.817

Significant PR (95% CI) and *p* trend <0.05 were highlighted in bold. Model 1: Adjusted for age and sex. Model 2: Model 1 + Smoking history + Alcohol consumption + Education + Physical activity + Depression + Living place. Sensitivity 1: Participants in all three waves of the FFQ in 2011, 2012, and 2013. Sensitivity 2: Model 2 participants without a history of hypertension (for hypertension), diabetes (for fasting blood glucose and impaired glucose control), hyperlipidemia (for TC, LDL-C, HDL-C, and triglycerides), and the three together (for MetS and overweight). PR, prevalence ratio; CI, confidence interval; FHMS, Fukushima Health Management Survey; BMI, body mass index; FFQ, food frequency survey; TC, total cholesterol; LDL-C, low-density lipoprotein cholesterol; HDL-C, high-density lipoprotein cholesterol; MetS, metabolic syndrome.

**Table 5:** Prevalence ratios (PRs, 95% CIs) for cardiometabolic factors in 2015 by quartiles of dietary pattern scores during 2011–2013, FHMS.

	BMI ≥ 25 (kg/m^2^)	Hypertension	Fasting Blood Glucose ≥ 126 (mg/dL)	HbA1c1 ≥ 6.5%	TC ≥ 220 (mg/dL)	LDL-C ≥ 140 (mg/dL)	HDL-C < 40 (mg/dL)	Triglycerides ≥ 150 (mg/dL)	MetS
**Vegetable**									
Model 1	1.00 (ref.)	1.00 (ref.)	1.00 (ref.)	1.00 (ref.)	1.00 (ref.)	1.00 (ref.)	1.00 (ref.)	1.00 (ref.)	1.00 (ref.)
	0.99 (0.97–1.01)	1.00 (0.98–1.01)	1.00 (0.99–1.01)	1.00 (0.99–1.01)	1.00 (0.99–1.02)	0.99 (0.97–1.00)	**0.99 (0.98–1.00)**	**0.98 (0.97–0.99)**	1.00 (0.98–1.01)
	**0.98 (0.96–0.99)**	0.99 (0.98–1.01)	1.00 (0.98–1.01)	1.00 (0.98–1.01)	0.99 (0.97–1.00)	**0.97 (0.96–0.99)**	**0.99 (0.98–1.00)**	**0.97 (0.96–0.99)**	**0.98 (0.96–1.00)**
	**0.95 (0.93–0.97)**	**0.97 (0.96–0.99)**	1.00 (0.98–1.01)	0.99 (0.98–1.01)	**0.97 (0.96–0.99)**	**0.96 (0.94–0.98)**	**0.98 (0.97–0.99)**	**0.95 (0.94–0.97)**	**0.97 (0.95–0.99)**
*p* trend	**<0.0001**	**0.0001**	0.396	0.262	**<0.0001**	**<0.0001**	**<0.0001**	**<0.0001**	**<0.0001**
Model 2	1.00 (ref.)	1.00 (ref.)	1.00 (ref.)	1.00 (ref.)	1.00 (ref.)	1.00 (ref.)	1.00 (ref.)	1.00 (ref.)	1.00 (ref.)
	0.99 (0.98–1.01)	1.00 (0.99–1.01)	1.00 (0.99–1.01)	1.00 (0.99–1.01)	1.00 (0.99–1.02)	0.99 (0.97–1.00)	0.99 (0.98–1.00)	**0.98 (0.97–1.00)**	1.00 (0.98–1.01)
	**0.98 (0.96–1.00)**	0.99 (0.98–1.01)	1.00 (0.98–1.01)	0.99 (0.98–1.01)	0.99 (0.98–1.00)	**0.97 (0.96–0.99)**	0.99 (0.98–1.00)	**0.98 (0.97–1.00)**	0.99 (0.97–1.00)
	**0.96 (0.94–0.98)**	**0.98 (0.96–0.99)**	0.99 (0.98–1.01)	0.99 (0.98–1.00)	**0.98 (0.96–0.99)**	**0.96 (0.95–0.98)**	**0.99 (0.98–1.00)**	**0.96 (0.95–0.98)**	**0.98 (0.96–0.99)**
*p* trend	**<0.0001**	**0.001**	0.382	0.156	**0.0004**	**<0.0001**	**0.034**	**<0.0001**	**0.003**
Sensitivity 1	1.00 (ref.)	1.00 (ref.)	1.00 (ref.)	1.00 (ref.)	1.00 (ref.)	1.00 (ref.)	1.00 (ref.)	1.00 (ref.)	1.00 (ref.)
	0.99 (0.97–1.02)	0.98 (0.97–1.00)	1.01 (1.00–1.02)	1.00 (0.99–1.02)	1.00 (0.98–1.02)	**0.98 (0.96–1.00)**	0.99 (0.98–1.00)	**0.97 (0.95–0.99)**	0.99 (0.97–1.01)
	0.99 (0.96–1.01)	**0.98 (0.96–1.00)**	1.00 (0.99–1.02)	1.00 (0.98–1.01)	0.99 (0.97–1.01)	**0.97 (0.95–0.99)**	1.00 (0.98–1.01)	**0.98 (0.96–1.00)**	0.99 (0.97–1.01)
	**0.96 (0.94–0.98)**	**0.97 (0.95–0.99)**	1.00 (0.98–1.02)	1.00 (0.98–1.02)	**0.97 (0.95–0.99)**	**0.96 (0.94–0.98)**	**0.98 (0.96–0.99)**	**0.96 (0.94–0.98)**	**0.97 (0.95–0.99)**
*p* trend	**0.0004**	**0.003**	0.739	0.896	**0.001**	**0.001**	**0.006**	**0.0002**	**0.011**
Sensitivity 2	1.00 (ref.)	1.00 (ref.)	1.00 (ref.)	1.00 (ref.)	1.00 (ref.)	1.00 (ref.)	1.00 (ref.)	1.00 (ref.)	1.00 (ref.)
	1.00 (0.97–1.02)	1.01 (0.99–1.02)	0.99 (0.99–1.00)	1.00 (0.99–1.00)	1.01 (0.99–1.02)	0.99 (0.98–1.01)	1.00 (0.98–1.01)	0.99 (0.97–1.00)	1.00 (0.98–1.01)
	0.98 (0.95–1.00)	1.00 (0.99–1.02)	**0.99 (0.98–1.00)**	0.99 (0.99–1.00)	0.99 (0.98–1.01)	**0.98 (0.96–1.00)**	0.99 (0.98–1.01)	**0.98 (0.96–1.00)**	0.99 (0.97–1.00)
	0.98 (0.95–1.00)	1.01 (1.00–1.03)	**0.99 (0.98–1.00)**	**0.99 (0.98–1.00)**	**0.98 (0.96–1.00)**	**0.96 (0.94–0.98)**	**0.99 (0.97–1.00)**	**0.97 (0.95–0.98)**	0.98 (0.97–1.00)
*p* trend	**0.03**	0.203	**0.02**	**0.028**	**0.008**	**<0.0001**	**0.026**	**0.0002**	**0.035**
**Juice/Milk**									
Model 1	1.00 (ref.)	1.00 (ref.)	1.00 (ref.)	1.00 (ref.)	1.00 (ref.)	1.00 (ref.)	1.00 (ref.)	1.00 (ref.)	1.00 (ref.)
	0.99 (0.98–1.01)	**0.99 (0.97–1.00)**	1.00 (0.99–1.01)	1.01 (1.00–1.02)	1.00 (0.99–1.02)	1.01 (0.99–1.02)	1.00 (0.99–1.01)	1.00 (0.98–1.01)	1.00 (0.98–1.01)
	**0.98 (0.97–1.00)**	**0.97 (0.96–0.99)**	1.01 (0.99–1.02)	1.01 (1.00–1.02)	1.01 (1.00–1.02)	**1.02 (1.00–1.03)**	1.00 (0.99–1.01)	1.00 (0.98–1.01)	1.00 (0.99–1.02)
	0.99 (0.98–1.01)	**0.97 (0.95–0.98)**	1.00 (0.99–1.01)	**1.02 (1.00–1.03)**	**1.03 (1.01–1.04)**	**1.03 (1.01–1.04)**	0.99 (0.98–1.00)	1.00 (0.98–1.01)	1.01 (0.99–1.02)
*p* trend	0.299	**<0.0001**	0.607	**0.008**	**0.0002**	**0.001**	0.243	0.751	0.285
Model 2	1.00 (ref.)	1.00 (ref.)	1.00 (ref.)	1.00 (ref.)	1.00 (ref.)	1.00 (ref.)	1.00 (ref.)	1.00 (ref.)	1.00 (ref.)
	1.00 (0.98–1.01)	0.99 (0.97–1.00)	1.00 (0.99–1.02)	1.01 (1.00–1.02)	1.01 (0.99–1.02)	1.01 (0.99–1.03)	1.00 (0.99–1.01)	1.00 (0.99–1.02)	1.00 (0.98–1.02)
	0.99 (0.97–1.00)	**0.98 (0.96–0.99)**	1.01 (0.99–1.02)	1.01 (1.00–1.02)	1.01 (1.00–1.03)	**1.02 (1.00–1.04)**	1.00 (0.99–1.01)	1.00 (0.99–1.02)	1.01 (0.99–1.02)
	1.00 (0.98–1.02)	**0.97 (0.96–0.98)**	1.00 (0.99–1.01)	**1.01 (1.00–1.02)**	**1.03 (1.02–1.04)**	**1.03 (1.01–1.05)**	1.00 (0.99–1.01)	1.01 (0.99–1.02)	1.01 (1.00–1.03)
*p* trend	0.898	**<0.0001**	0.594	**0.029**	**<0.0001**	**0.0003**	0.397	0.265	0.086
Sensitivity 1	1.00 (ref.)	1.00 (ref.)	1.00 (ref.)	1.00 (ref.)	1.00 (ref.)	1.00 (ref.)	1.00 (ref.)	1.00 (ref.)	1.00 (ref.)
	1.00 (0.98–1.02)	1.00 (0.98–1.01)	1.01 (1.00–1.03)	1.00 (0.99–1.02)	1.00 (0.98–1.02)	1.01 (0.99–1.03)	1.00 (0.98–1.01)	0.99 (0.97–1.01)	1.02 (0.99–1.04)
	0.98 (0.96–1.00)	**0.98 (0.97–1.00)**	1.01 (0.99–1.02)	1.01 (0.99–1.02)	1.01 (0.99–1.03)	**1.02 (1.00–1.04)**	1.00 (0.99–1.02)	1.00 (0.98–1.01)	1.02 (0.99–1.04)
	1.00 (0.98–1.02)	0.99 (0.97–1.00)	1.01 (0.99–1.02)	1.01 (1.00–1.03)	**1.02 (1.00–1.04)**	**1.03 (1.01–1.05)**	0.99 (0.98–1.01)	1.00 (0.98–1.02)	**1.03 (1.01–1.05)**
*p* trend	0.767	0.087	0.637	0.158	**0.004**	**0.005**	0.467	0.81	**0.021**
Sensitivity 2	1.00 (ref.)	1.00 (ref.)	1.00 (ref.)	1.00 (ref.)	1.00 (ref.)	1.00 (ref.)	1.00 (ref.)	1.00 (ref.)	1.00 (ref.)
	0.99 (0.97–1.01)	0.99 (0.97–1.01)	1.00 (0.99–1.00)	1.00 (0.99–1.01)	1.01 (1.00–1.03)	1.02 (1.00–1.03)	1.00 (0.99–1.01)	1.01 (0.99–1.03)	1.00 (0.99–1.02)
	0.99 (0.97–1.01)	0.99 (0.97–1.00)	1.00 (0.99–1.00)	1.00 (0.99–1.01)	**1.02 (1.01–1.04)**	**1.03 (1.01–1.05)**	1.01 (0.99–1.02)	1.01 (0.99–1.02)	1.01 (0.99–1.02)
	1.01 (0.99–1.04)	0.98 (0.97–1.00)	1.00 (0.99–1.01)	1.00 (0.99–1.01)	**1.05 (1.03–1.06)**	**1.05 (1.03–1.07)**	0.99 (0.98–1.00)	1.01 (0.99–1.03)	1.00 (0.99–1.02)
*p* trend	0.27	0.097	0.785	0.587	**<0.0001**	**<0.0001**	0.364	0.421	0.793
**Meat**									
Model 1	1.00 (ref.)	1.00 (ref.)	1.00 (ref.)	1.00 (ref.)	1.00 (ref.)	1.00 (ref.)	1.00 (ref.)	1.00 (ref.)	1.00 (ref.)
	1.00 (0.99–1.02)	1.00 (0.98–1.01)	0.99 (0.98–1.00)	0.99 (0.98–1.00)	**1.02 (1.01–1.04)**	1.01 (0.99–1.02)	0.99 (0.98–1.00)	1.00 (0.99–1.02)	0.99 (0.98–1.01)
	0.99 (0.97–1.01)	0.99 (0.98–1.00)	1.00 (0.99–1.01)	1.00 (0.99–1.01)	**1.01 (1.00–1.03)**	1.01 (1.00–1.03)	0.99 (0.98–1.00)	0.99 (0.98–1.01)	0.99 (0.98–1.01)
	1.00 (0.99–1.02)	0.99 (0.97–1.00)	1.00 (0.99–1.01)	1.00 (0.99–1.01)	1.00 (0.98–1.01)	1.01 (0.99–1.02)	**0.99 (0.98–1.00)**	**0.98 (0.97–1.00)**	1.00 (0.98–1.01)
*p* trend	0.953	0.1	0.93	0.999	0.303	0.305	**0.008**	**0.017**	0.758
Model 2	1.00 (ref.)	1.00 (ref.)	1.00 (ref.)	1.00 (ref.)	1.00 (ref.)	1.00 (ref.)	1.00 (ref.)	1.00 (ref.)	1.00 (ref.)
	1.01 (0.99–1.02)	1.00 (0.98–1.01)	0.99 (0.98–1.00)	0.99 (0.98–1.00)	**1.02 (1.00–1.03)**	1.00 (0.99–1.02)	0.99 (0.98–1.00)	1.00 (0.99–1.02)	1.00 (0.98–1.01)
	0.99 (0.98–1.01)	0.99 (0.98–1.01)	1.00 (0.99–1.01)	1.00 (0.99–1.01)	1.01 (1.00–1.03)	1.01 (1.00–1.03)	0.99 (0.98–1.00)	0.99 (0.98–1.01)	1.00 (0.98–1.01)
	1.01 (0.99–1.02)	0.99 (0.98–1.01)	1.00 (0.99–1.01)	1.00 (0.99–1.01)	1.00 (0.98–1.01)	1.01 (0.99–1.02)	**0.99 (0.98–1.00)**	**0.98 (0.97–1.00)**	1.00 (0.98–1.02)
*p* trend	0.593	0.21	0.943	0.91	0.211	0.368	**0.008**	**0.017**	0.957
Sensitivity 1	1.00 (ref.)	1.00 (ref.)	1.00 (ref.)	1.00 (ref.)	1.00 (ref.)	1.00 (ref.)	1.00 (ref.)	1.00 (ref.)	1.00 (ref.)
	1.00 (0.98–1.02)	0.99 (0.97–1.01)	1.00 (0.99–1.02)	1.00 (0.99–1.01)	**1.02 (1.00–1.04)**	1.01 (0.99–1.03)	1.00 (0.98–1.01)	1.00 (0.98–1.02)	1.00 (0.98–1.02)
	0.99 (0.97–1.01)	0.99 (0.97–1.01)	**1.02 (1.00–1.03)**	1.01 (0.99–1.02)	1.01 (0.99–1.03)	1.01 (0.99–1.04)	0.99 (0.98–1.01)	0.99 (0.97–1.01)	1.00 (0.98–1.02)
	1.01 (0.99–1.030)	0.99 (0.98–1.01)	1.00 (0.98–1.01)	1.00 (0.98–1.01)	0.99 (0.98–1.01)	1.01 (0.99–1.03)	**0.99 (0.97–1.00)**	0.98 (0.97–1.00)	1.01 (0.99–1.03)
*p* trend	0.457	0.634	0.97	0.854	0.205	0.402	**0.035**	0.058	0.484
Sensitivity 2	1.00 (ref.)	1.00 (ref.)	1.00 (ref.)	1.00 (ref.)	1.00 (ref.)	1.00 (ref.)	1.00 (ref.)	1.00 (ref.)	1.00 (ref.)
	1.00 (0.98–1.02)	1.00 (0.98–1.02)	0.99 (0.98–1.00)	1.00 (0.99–1.00)	1.01 (1.00–1.03)	1.00 (0.98–1.02)	1.00 (0.99–1.01)	1.00 (0.99–1.02)	1.01 (0.99–1.02)
	1.00 (0.97–1.02)	1.00 (0.98–1.02)	0.99 (0.98–1.00)	1.00 (0.99–1.00)	1.01 (0.99–1.03)	1.01 (0.99–1.03)	0.99 (0.98–1.01)	1.00 (0.98–1.01)	1.00 (0.99–1.02)
	1.01 (0.99–1.04)	1.01 (0.99–1.030)	0.99 (0.98–1.00)	0.99 (0.99–1.00)	0.99 (0.97–1.00)	1.00 (0.98–1.02)	**0.98 (0.97–1.00)**	0.99 (0.97–1.00)	1.00 (0.99–1.01)
*p* trend	0.358	0.256	0.147	0.148	**0.041**	0.923	**0.002**	0.083	0.834

Significant PR (95% CI) and *p* trend were highlighted in bold. Model 1: Adjusted for age and sex. Model 2: Model 1+ Smoking history + Alcohol consumption + Education + Physical activity + Depression + Living place. Sensitivity 1: Participants in all three waves of food frequency survey in 2011, 2012, and 2013. Sensitivity 2: Model 2 participants without a history of hypertension (for hypertension), diabetes (for fasting blood glucose and impaired glucose control), hyperlipidemia (for TC, LDL-C, HDL-C, and triglycerides), and the three together (for MetS and overweight). PR, prevalence ratio; CI, confidence interval; FHMS, Fukushima Health Management Survey; BMI, body mass index; FFQ, food frequency survey; TC, total cholesterol; LDL-C, low-density lipoprotein cholesterol; HDL-C, high-density lipoprotein cholesterol; MetS, metabolic syndrome.

## Supplementary Materials

The following are available online at https://www.mdpi.com/2072-6643/12/1/129/s1, Figure S1. Flowchart for 2014 and 2015 study participants, Fukushima Health Management Survey. Table S1. Cumulative mean scores of dietary patterns during 2011–2013 by cardiometabolic factors in 2014, Fukushima Health Management Survey. Table S2. Cumulative mean scores of dietary patterns during 2011–2013 by cardiometabolic factors in 2015, Fukushima Health Management Survey.

## References

[1] Popkin B.M. (2006). Global nutrition dynamics: The world is shifting rapidly toward a diet linked with noncommunicable diseases. Am. J. Clin. Nutr..

[2] Ohira T., Nakano H., Nagai M., Yumiya Y., Zhang W., Uemura M., Sakai A., Hashimoto S., Fukushima Health Management Survey Group (2017). Changes in Cardiovascular Risk Factors after the Great East Japan Earthquake. Asia Pac. J. Public Health.

[3] Takahashi S., Nakamura M., Yonekura Y., Tanno K., Sakata K., Ogawa A., Kobayashi S. (2016). Association between relocation and changes in cardiometabolic risk factors: A longitudinal study in tsunami survivors of the 2011 Great East Japan Earthquake. BMJ Open.

[4] Nagai M., Ohira T., Takahashi H., Nakano H., Sakai A., Hashimoto S., Yasumura S., Abe M. (2018). Impact of evacuation onstrends in the prevalence, treatment, and control of hypertension before and after a disaster. J. Hypertens..

[5] Hu F.B. (2002). Dietary pattern analysis: A new direction in nutritional epidemiology. Curr. Opin. Lipidol..

[6] Borges C.A., Rinaldi A.E., Conde W.L., Mainardi G.M., Behar D., Slater B. (2015). Dietary patterns: A literature review of the methodological characteristics of the main step of the multivariate analyzes. Rev. Bras. Epidemiol..

[7] Wesolowska E., Jankowska A., Trafalska E., Kaluzny P., Grzesiak M., Dominowska J., Hanke W., Calamandrei G., Polanska K. (2019). Sociodemographic, Lifestyle, Environmental and Pregnancy-Related Determinants of Dietary Patterns during Pregnancy. Int. J. Environ. Res Public Health.

[8] Yokoyama Y., Barnard N.D., Levin S.M., Watanabe M. (2014). Vegetarian diets and glycemic control in diabetes: A systematic review and meta-analysis. Cardiovasc. Diagn. Ther..

[9] Akhlaghi M. (2019). Dietary Approaches to Stop Hypertension (DASH): Potential mechanisms of action against risk factors of the metabolic syndrome. Nutr. Res. Rev..

[10] Okada E., Takahashi K., Nakamura K., Ukawa S., Takabayashi S., Nakamura M., Sasaki S., Tamakoshi A., Takimoto H. (2019). Dietary patterns and abnormal glucose tolerance among Japanese: Findings from the National Health and Nutrition Survey, 2012. Public Health Nutr..

[11] Na L., Han T., Zhang W., Wu X., Na G., Du S., Li Y., Sun C. (2015). A Snack Dietary Pattern Increases the Risk of Hypercholesterolemia in Northern Chinese Adults: A Prospective Cohort Study. PLoS ONE.

[12] Godos J., Zappala G., Bernardini S., Giambini I., Bes-Rastrollo M., Martinez-Gonzalez M. (2017). Adherence to the Mediterranean diet is inversely associated with metabolic syndrome occurrence: A meta-analysis of observational studies. Int. J. Food Sci. Nutr..

[13] Mattioli A.V., Palmiero P., Manfrini O., Puddu P.E., Nodari S., Dei Cas A., Mercuro G., Scrutinio D., Palermo P., Sciomer S. (2017). Mediterranean diet impact on cardiovascular diseases: A narrative review. J. Cardiovasc. Med..

[14] Asghari G., Yuzbashian E., Mirmiran P., Hooshmand F., Najafi R., Azizi F. (2016). Dietary Approaches to Stop Hypertension (DASH) Dietary Pattern Is Associated with Reduced Incidence of Metabolic Syndrome in Children and Adolescents. J. Pediatr..

[15] Sadakane A., Tsutsumi A., Gotoh T., Ishikawa S., Ojima T., Kario K., Nakamura Y., Kayaba K. (2008). Dietary patterns and levels of blood pressure and serum lipids in a Japanese population. J. Epidemiol..

[16] Htun N.C., Suga H., Imai S., Shimizu W., Ishikawa-Takata K., Takimoto H. (2018). Dietary pattern and its association with blood pressure and blood lipid profiles among Japanese adults in the 2012 Japan National Health and Nutrition Survey. Asia Pac. J. Clin. Nutr..

[17] Iwasaki Y., Arisawa K., Katsuura-Kamano S., Uemura H., Tsukamoto M., Kadomatsu Y., Okada R., Hishida A., Tanaka K., Hara M. (2019). Associations of Nutrient Patterns with the Prevalence of Metabolic Syndrome: Results from the Baseline Data of the Japan Multi-Institutional Collaborative Cohort Study. Nutrients.

[18] Yasumura S., Hosoya M., Yamashita S., Kamiya K., Abe M., Akashi M., Kodama K., Ozasa K., Fukushima Health Management Survey Group (2012). Study protocol for the Fukushima Health Management Survey. J. Epidemiol..

[19] Sauvaget C., Allen N., Hayashi M., Spencer E., Nagano J. (2002). Validation of a food frequency questionnaire in the Hiroshima/Nagasaki Life Span Study. J. Epidemiol..

[20] Zhang W., Ohira T., Abe M., Kamiya K., Yamashita S., Yasumura S., Ohtsuru A., Masaharu M., Harigane M., Horikoshi N. (2017). Evacuation after the Great East Japan Earthquake was associated with poor dietary intake: The Fukushima Health Management Survey. J. Epidemiol..

[21] Luger E., Aspalter R., Luger M., Longin R., Rieder A., Dorner T.E. (2016). Changes of dietary patterns during participation in a web-based weight-reduction programme. Public Health Nutr..

[22] Lin J., Fung T.T., Hu F.B., Curhan G.C. (2011). Association of dietary patterns with albuminuria and kidney function decline in older white women: A subgroup analysis from the Nurses’ Health Study. Am. J. Kidney Dis..

[23] Shi Z., Taylor A.W., Riley M., Byles J., Liu J., Noakes M. (2018). Association between dietary patterns, cadmium intake and chronic kidney disease among adults. Clin. Nutr..

[24] Tomata Y., Sugiyama K., Kaiho Y., Honkura K., Watanabe T., Zhang S., Sugawara Y., Tsuji I. (2016). Dietary Patterns and Incident Dementia in Elderly Japanese: The Ohsaki Cohort 2006 Study. J. Gerontol. Ser. A Biol. Sci. Med. Sci..

[25] Maruyama K., Iso H., Date C., Kikuchi S., Watanabe Y., Wada Y., Inaba Y., Tamakoshi A., JACC Study Group (2013). Dietary patterns and risk of cardiovascular deaths among middle-aged Japanese: JACC Study. Nutr. Metab. Cardiovasc. Dis..

[26] Murakami K., Shinozaki N., Fujiwara A., Yuan X., Hashimoto A., Fujihashi H., Wang H.C., Livingstone M.B.E., Sasaki S. (2019). A Systematic Review of Principal Component Analysis-Derived Dietary Patterns in Japanese Adults: Are Major Dietary Patterns Reproducible Within a Country?. Adv. Nutr..

[27] Nanri A., Yoshida D., Yamaji T., Mizoue T., Takayanagi R., Kono S. (2008). Dietary patterns and C-reactive protein in Japanese men and women. Am. J. Clin. Nutr..

[28] Ito T., Kawakami R., Tanisawa K., Miyawaki R., Ishii K., Torii S., Suzuki K., Sakamoto S., Muraoka I., Oka K. (2019). Dietary patterns and abdominal obesity in middle-aged and elderly Japanese adults: Waseda Alumni’s Sports, Exercise, Daily Activity, Sedentariness and Health Study (WASEDA’S Health Study). Nutrition.

[29] Lopez-Garcia E., Schulze M.B., Fung T.T., Meigs J.B., Rifai N., Manson J.E., Hu F.B. (2004). Major dietary patterns are related to plasma concentrations of markers of inflammation and endothelial dysfunction. Am. J. Clin. Nutr..

[30] Fung T.T., Rimm E.B., Spiegelman D., Rifai N., Tofler G.H., Willett W.C., Hu F.B. (2001). Association between dietary patterns and plasma biomarkers of obesity and cardiovascular disease risk. Am. J. Clin. Nutr..

[31] Esmaillzadeh A., Kimiagar M., Mehrabi Y., Azadbakht L., Hu F.B., Willett W.C. (2007). Dietary patterns, insulin resistance, and prevalence of the metabolic syndrome in women. Am. J. Clin. Nutr..

[32] Yamori Y., Sagara M., Arai Y., Kobayashi H., Kishimoto K., Matsuno I., Mori H., Mori M. (2017). Soy and fish as features of the Japanese diet and cardiovascular disease risks. PLoS ONE.

[33] Suzuki N., Goto Y., Ota H., Kito K., Mano F., Joo E., Ikeda K., Inagaki N., Nakayama T. (2018). Characteristics of the Japanese Diet Described in Epidemiologic Publications: A Qualitative Systematic Review. J. Nutr. Sci. Vitaminol..

[34] Bahari T., Uemura H., Katsuura-Kamano S., Yamaguchi M., Nakamoto M., Miki K., Ishizu M., Arisawa K. (2018). Nutrient-Derived Dietary Patterns and Their Association with Metabolic Syndrome in a Japanese Population. J. Epidemiol..

[35] Mizoue T., Yamaji T., Tabata S., Yamaguchi K., Ogawa S., Mineshita M., Kono S. (2006). Dietary patterns and glucose tolerance abnormalities in Japanese men. J. Nutr..

[36] Tormo M.J., Navarro C., Chirlaque M.D., Barber X., EPIC Group of Spain (2000). European Prospective Investigation on Cancer. Is there a different dietetic pattern depending on self-knowledge of high blood pressure?. Eur. J. Epidemiol..

[37] Okada E., Takahashi K., Takimoto H., Takabayashi S., Kishi T., Kobayashi T., Nakamura K., Ukawa S., Nakamura M., Sasaki S. (2018). Dietary patterns among Japanese adults: Findings from the National Health and Nutrition Survey, 2012. Asia Pac. J. Clin. Nutr..

[38] Lee K.W., Woo H.D., Cho M.J., Park J.K., Kim S.S. (2019). Identification of Dietary Patterns Associated with Incidence of Hyperglycemia in Middle-Aged and Older Korean Adults. Nutrients.

[39] Li M., Shi Z. (2017). Dietary Pattern during 1991–2011 and Its Association with Cardio Metabolic Risks in Chinese Adults: The China Health and Nutrition Survey. Nutrients.

[40] Hermansen K., Sondergaard M., Hoie L., Carstensen M., Brock B. (2001). Beneficial effects of a soy-based dietary supplement on lipid levels and cardiovascular risk markers in type 2 diabetic subjects. Diabetes Care.

[41] Olinto M.T., Gigante D.P., Horta B., Silveira V., Oliveira I., Willett W. (2012). Major dietary patterns and cardiovascular risk factors among young Brazilian adults. Eur. J. Nutr..

[42] Nanri H., Nakamura K., Hara M., Higaki Y., Imaizumi T., Taguchi N., Sakamoto T., Horita M., Shinchi K., Tanaka K. (2011). Association between dietary pattern and serum C-reactive protein in Japanese men and women. J. Epidemiol..

[43] Na W., Chung B., Sohn C. (2019). A Relationship between Dietary Patterns and Dyslipidemia in Urban-dwelling Middle-Aged Korean Men: Using Korean Genome and Epidemiology Study (KoGES). Clin. Nutr. Res..

[44] Tomata Y., Zhang S., Kaiho Y., Tanji F., Sugawara Y., Tsuji I. (2019). Nutritional characteristics of the Japanese diet: A cross-sectional study of the correlation between Japanese Diet Index and nutrient intake among community-based elderly Japanese. Nutrition.

[45] Iso H. (2011). Lifestyle and cardiovascular disease in Japan. J. Atheroscler. Thromb..

[46] Lamb K.E., Olstad D.L., Nguyen C., Milte C., McNaughton S.A. (2017). Missing data in FFQs: Making assumptions about item non-response. Public Health Nutr..

[47] Yasumura S., Abe M. (2017). Fukushima Health Management Survey and Related Issues. Asia Pac. J. Public Health.

[48] Tsuboyama-Kasaoka N., Purba M.B. (2014). Nutrition and earthquakes: Experience and recommendations. Asia Pac. J. Clin. Nutr..

[49] McCann S.E., Marshall J.R., Brasure J.R., Graham S., Freudenheim J.L. (2001). Analysis of patterns of food intake in nutritional epidemiology: Food classification in principal components analysis and the subsequent impact on estimates for endometrial cancer. Public Health Nutr..

[50] Tomata Y., Watanabe T., Sugawara Y., Chou W.T., Kakizaki M., Tsuji I. (2014). Dietary patterns and incident functional disability in elderly Japanese: The Ohsaki Cohort 2006 study. J. Gerontol. Ser. A Biol. Sci. Med. Sci..

