# Supplementary material for: Associations between Dietary Patterns and Cardiometabolic Risks in Japan: A Cross-Sectional Study from the Fukushima Health Management Survey, 2011–2015
Source: Nutrients. 2020 Jan 2;12(1):129. doi: 10.3390/nu12010129 (PMC7019971; doi:10.3390/nu12010129)
Supplement: Supplementary file 1 [file nutrients-12-00129-s001.docx]

Subjects attended the Mental Health Survey, 2011, 2012, or 2013, aged ≥16 years

n = 172,664

Examine the associations between means of accumulative dietary pattern scores and health checkup outcomes.

n = 15,409

Subjects whose FFQ of had ≥3 missing food items, n = 2232

Pregnant women, n = 1793

Current or history of stroke, heart disease, or cancer, n = 33,269

Examine the associations between accumulative dietary pattern scores and health checkup outcomes.

n = 14,999

Subjects, n = 137,602

Subjects had FFQ of

≥3 missing food items, n = 2425

Subjects (n = 18,173) of health checkup in 2014 had FFQs^*^ in 2011, 2012, or 2013.

n = 36,996

Subjects (n = 17,973) of health checkup in 2015 had FFQs in 2011, 2012, or 2013.

n = 35,875

Derive dietary patterns and compute accumulated dietary pattern scores.

n = 33,643

Derive dietary patterns and compute accumulated dietary pattern scores.

n = 34,571

**Figure S1.** Flowchart for 2014 and 2015 study participants, Fukushima Health Management Survey. FFQ, food frequency questionnaire

| **Table S1.** Cumulative mean score of dietary patterns during 2011-2013 by cardiometabolic factors in 2014, Fukushima Health Management Survey | | | | | | | | | |
| --- | --- | --- | --- | --- | --- | --- | --- | --- | --- |
|  | n | Vegetable | |  | Juice/milk | |  | Meat | |
|  |  | Median (IQR) | *P-value* |  | Median (IQR) | *P-value* |  | Median (IQR) | *P-value* |
| BMI ≥25 (kg/m^2^) |  |  | 0.004 |  |  | 0.019 |  |  | <0.001 |
| No | 7329 | 0 (-0.65, 0.62) |  |  | -0.14 (-0.64, 0.46) |  |  | -0.14 (-0.6-0.44) |  |
| Yes | 3173 | -0.07 (-0.7, 0.55) |  |  | -0.18 (-0.65, 0.43) |  |  | -0.2 (-0.62-0.39) |  |
| Hypertension |  |  | <0.001 |  |  | 0.836 |  |  | <0.001 |
| No | 5988 | -0.09 (-0.75, 0.55) |  |  | -0.15 (-0.63, 0.47) |  |  | -0.1 (-0.56-0.5) |  |
| Yes | 4514 | 0.06 (-0.56, 0.67) |  |  | -0.17 (-0.66, 0.43) |  |  | -0.26 (-0.66-0.32) |  |
| Fasting blood glucose ≥126(mg/dl) |  |  | 0.005 |  |  | 0.487 |  |  | 0.001 |
| No | 9779 | -0.03 (-0.66, 0.6) |  |  | -0.15 (-0.64, 0.45) |  |  | -0.16 (-0.6-0.43) |  |
| Yes | 723 | 0.06 (-0.64, 0.68) |  |  | -0.16 (-0.68, 0.43) |  |  | -0.2 (-0.63-0.33) |  |
| Hemoglobin A1c1 ≥6.5% |  |  | 0.001 |  |  | 0.122 |  |  | <0.001 |
| No | 9791 | -0.03 (-0.66, 0.6) |  |  | -0.16 (-0.64, 0.44) |  |  | -0.16 (-0.6-0.43) |  |
| Yes | 711 | 0.06 (-0.65, 0.65) |  |  | -0.1 (-0.61, 0.53) |  |  | -0.22 (-0.64-0.31) |  |
| TC ≥220(mg/dl) |  |  | 0.032 |  |  | <0.001 |  |  | 0.003 |
| No | 3850 | 0.01 (-0.64, 0.63) |  |  | -0.18 (-0.66, 0.39) |  |  | -0.14 (-0.6-0.48) |  |
| Yes | 6652 | -0.05 (-0.68, 0.58) |  |  | -0.14 (-0.63, 0.48) |  |  | -0.18 (-0.61-0.39) |  |
| LDL-C ≥140(mg/dl) |  |  | <0.001 |  |  | 0.002 |  |  | 0.384 |
| No | 7351 | 0 (-0.64, 0.63) |  |  | -0.16 (-0.66, 0.44) |  |  | -0.17 (-0.61-0.43) |  |
| Yes | 3151 | -0.09 (-0.73, 0.55) |  |  | -0.13 (-0.61, 0.47) |  |  | -0.14 (-0.59-0.43) |  |
| HDL-C <40(mg/dl) |  |  | 0.086 |  |  | <0.001 |  |  | <0.001 |
| No | 10022 | -0.02 (-0.66, 0.61) |  |  | -0.15 (-0.64, 0.46) |  |  | -0.16 (-0.6-0.43) |  |
| Yes | 480 | -0.1 (-0.75, 0.46) |  |  | -0.26 (-0.7, 0.31) |  |  | -0.23 (-0.65-0.33) |  |
| Triglycerides ≥150(mg/dl) |  |  | <0.001 |  |  | 0.007 |  |  | 0.016 |
| No | 8631 | 0 (-0.65, 0.63) |  |  | -0.14 (-0.63, 0.46) |  |  | -0.16 (-0.6-0.44) |  |
| Yes | 1871 | -0.12 (-0.76, 0.5) |  |  | -0.22 (-0.68, 0.41) |  |  | -0.2 (-0.61-0.37) |  |
| Metabolic syndrome |  |  | 0.085 |  |  | 0.03 |  |  | <0.001 |
| No | 9081 | -0.02 (-0.66, 0.61) |  |  | -0.15 (-0.64, 0.46) |  |  | -0.15 (-0.6-0.45) |  |
| Yes | 1421 | -0.05 (-0.66, 0.53) |  |  | -0.2 (-0.67, 0.41) |  |  | -0.27 (-0.64-0.28) |  |
| IQR: interquartile range. TC, total cholesterol; LDL-C, low-density lipoprotein cholesterol; HDL-C, high-density lipoprotein cholesterol. | | | | | | | | | |

| **Table S2.** Cumulative mean scores of dietary patterns during 2011-2013 by cardiometabolic factors in 2015, Fukushima Health Management Survey | | | | | | | | | |
| --- | --- | --- | --- | --- | --- | --- | --- | --- | --- |
|  | n | Vegetable | |  | Juice/milk | |  | Meat | |
|  |  | Median (IQR) | *P-value* |  | Median (IQR) | *P-value* |  | Median (IQR) | *P-value* |
| BMI ≥25 (kg/m^2^) |  |  | <0.001 |  |  | 0.003 |  |  | 0.02 |
| No | 7186 | -0.02 (-0.67, 0.61) |  |  | -0.14 (-0.65, 0.45) |  |  | -0.15 (-0.59-0.45) |  |
| Yes | 3115 | -0.15 (-0.74, 0.49) |  |  | -0.21 (-0.69, 0.42) |  |  | -0.17 (-0.6-0.44) |  |
| Hypertension |  |  | <0.001 |  |  | 0.004 |  |  | <0.001 |
| No | 5859 | -0.12 (-0.78, 0.53) |  |  | -0.12 (-0.64, 0.46) |  |  | -0.08 (-0.53-0.54) |  |
| Yes | 4442 | 0.02 (-0.6, 0.63) |  |  | -0.21 (-0.7, 0.4) |  |  | -0.25 (-0.66-0.33) |  |
| Fasting blood glucose ≥126(mg/dl) |  |  | 0.003 |  |  | 0.444 |  |  | <0.001 |
| No | 9609 | -0.06 (-0.7, 0.57) |  |  | -0.16 (-0.66, 0.44) |  |  | -0.15 (-0.59-0.45) |  |
| Yes | 692 | 0.01 (-0.65, 0.58) |  |  | -0.15 (-0.67, 0.41) |  |  | -0.2 (-0.66-0.35) |  |
| Hemoglobin A1c1 ≥6.5% |  |  | 0.007 |  |  | 0.036 |  |  | 0.002 |
| No | 9547 | -0.07 (-0.7, 0.57) |  |  | -0.17 (-0.67, 0.44) |  |  | -0.15 (-0.59-0.45) |  |
| Yes | 754 | 0.05 (-0.63, 0.58) |  |  | -0.11 (-0.62, 0.48) |  |  | -0.2 (-0.65-0.35) |  |
| TC ≥220(mg/dl) |  |  | 0.762 |  |  | <0.001 |  |  | 0.038 |
| No | 3837 | -0.06 (-0.71, 0.57) |  |  | -0.2 (-0.69, 0.38) |  |  | -0.13 (-0.6-0.49) |  |
| Yes | 6464 | -0.06 (-0.69, 0.57) |  |  | -0.14 (-0.65, 0.47) |  |  | -0.16 (-0.59-0.41) |  |
| LDL-C ≥140(mg/dl) |  |  | <0.001 |  |  | <0.001 |  |  | 0.189 |
| No | 7258 | -0.05 (-0.69, 0.59) |  |  | -0.18 (-0.67, 0.41) |  |  | -0.17 (-0.6-0.43) |  |
| Yes | 3043 | -0.11 (-0.74, 0.54) |  |  | -0.11 (-0.64, 0.49) |  |  | -0.12 (-0.58-0.46) |  |
| HDL-C <40(mg/dl) |  |  | 0.001 |  |  | <0.001 |  |  | <0.001 |
| No | 9838 | -0.06 (-0.69, 0.58) |  |  | -0.16 (-0.66, 0.45) |  |  | -0.15 (-0.59-0.46) |  |
| Yes | 463 | -0.19 (-0.85, 0.45) |  |  | -0.24 (-0.69, 0.29) |  |  | -0.27 (-0.65-0.26) |  |
| Triglycerides ≥150(mg/dl) |  |  | <0.001 |  |  | 0.009 |  |  | <0.001 |
| No | 8488 | -0.04 (-0.67, 0.59) |  |  | -0.15 (-0.65, 0.45) |  |  | -0.14 (-0.59-0.46) |  |
| Yes | 1813 | -0.16 (-0.83, 0.47) |  |  | -0.22 (-0.7, 0.4) |  |  | -0.21 (-0.62-0.38) |  |
| Metabolic syndrome |  |  | 0.013 |  |  | 0.082 |  |  | <0.001 |
| No | 8855 | -0.05 (-0.69, 0.59) |  |  | -0.16 (-0.66, 0.44) |  |  | -0.14 (-0.59-0.46) |  |
| Yes | 1446 | -0.12 (-0.72, 0.5) |  |  | -0.2 (-0.7, 0.42) |  |  | -0.25 (-0.66-0.33) |  |
| IQR: interquartile range. TC, total cholesterol; LDL-C, low-density lipoprotein cholesterol; HDL-C, high-density lipoprotein cholesterol. | | | | | | | | | |
